# Genomically-selected antifungal Bacillaceae strains improve wheat yield and baking quality

**DOI:** 10.1007/s00253-025-13544-9

**Published:** 2025-07-10

**Authors:** Alejo Casal, Fernán Oscar Gizzi, Sol Agostina Figueroa, Tomás Denis Petitti, Facundo Ferragutti, Jimena Gaido, Mariano Alberto Torres Manno, Gabriel Céccoli, Luciana Paoletti, Christopher Dunlap, Lucas Damián Daurelio, Martín Espariz

**Affiliations:** 1 Laboratorio de Biotecnología e Inocuidad de los Alimentos, Facultad de Ciencias Bioquímicas y Farmacéuticas (FCByF), Universidad Nacional de Rosario (UNR), Municipalidad de Granadero Baigorria, Sede Suipacha 590, Rosario, Argentina; 2 Laboratorio de Genética y Fisiología de Bacterias Lácticas, Instituto de Biología Molecular y Celular de Rosario (IBR), Consejo Nacional de Investigaciones Científicas y Tecnológicas (CONICET), Sede FCByF - UNR, Rosario, Santa Fe, Argentina; 3 Laboratorio de Biotecnología Acuática, FCByF, UNR, Centro Científico, Tecnológico y Educativo Acuario del Río Paraná, Av. Eduardo Carrasco y Cordiviola s/n, Rosario, Argentina; 4 Área Bioinformática, Departamento de Matemática y Estadística, FCByF, UNR, Rosario, Argentina; 5 Instituto Nacional de Tecnología Agropecuaria (INTA), Estación Experimental Agropecuaria, Oliveros, Argentina; 6 Laboratorio de Investigaciones en Fisiología y Biología Molecular Vegetal (LIFiBVe), ICiAgro Litoral, Universidad Nacional del Litoral (UNL), CONICET, Facultad de Ciencias Agrarias (FCA), Kreder 2805, Esperanza, 3080HOF Argentina; 7 Cátedra de Fisiología Vegetal, FCA, UNL, Esperanza, Santa Fe, Argentina; 8 TAXON Bioinformatics Solutions S.A., Ibarlucea, Santa Fe, Argentina; 9 Instituto de Procesos Biotecnológicos y Químicos (IPROBYQ), FCByF-UNR-CONICET, Rosario, Argentina; 10 United States Department of Agriculture, Crop Bioprotection Research Unit, National Center for Agricultural Utilization Research, Agricultural Research Service, 1815 North University Street, Peoria, 61604 IL USA; 11 CONICET, Rosario, Argentina

**Keywords:** Plant-growth promoting rhizobacteria, *Priestia megaterium*, *Bacillus velezensis*, Comparative genomics, Gene mining, Field assays, Grain wheat quality

## Abstract

**Abstract:**

Soil microbial diversity degradation through agricultural intensification necessitates sustainable alternatives. This study employed genomic and phenotypic approaches to characterize wheat rhizosphere-associated Bacillaceae for agricultural applications. Initial screening of 576 sporulating isolates for antifungal activity against *Fusarium graminearum*, followed by RAPD analysis, identified 39 distinct genetic profiles, out of which 15 were classified in *Bacillus amyloliquefaciens* or *Priestia megaterium* groups by 16S RNA sequence. Whole-genome sequencing of selected strains enabled precise taxonomic classification and comprehensive trait prediction using in silico tools. Genomic mining revealed strain-specific distributions of beneficial traits, including antimicrobial compound production pathways and plant growth-promoting characteristics. Phenotypic validation confirmed key predicted traits while uncovering additional functionalities not detected in silico. Integration of kernel bioassays, pot experiments, and field trials identified *Bacillus velezensis* ZAV-W70 and *P. megaterium* ZAV-W64 as promising biofertilizer and biocontrol candidates, demonstrating enhanced yield without fungicides and improved bread-making quality, respectively. These findings highlight the value of combining genomic analysis with traditional screening methods for developing effective agricultural biologicals, contributing to sustainable wheat production practices.

**Key points:**

• *Rhizosphere Bacillaceae strains show dual plant growth promotion and biocontrol*

• *B. velezensis ZAV-W70 and P. megaterium ZAV-W64 increase wheat yield*

• *ZAV-W64 increases bread-making quality including total gluten and alveograph W*

**Supplementary Information:**

The online version contains supplementary material available at 10.1007/s00253-025-13544-9.

## Introduction

Members of the Bacillaceae family are integral components of soil microbial communities, playing a pivotal role in agro-biotechnological applications due to their diverse plant-beneficial effects. As prominent plant growth-promoting rhizobacteria (PGPR), they are widely utilized in agriculture and horticulture to enhance crop productivity and resilience. Key species, such as *Priestia megaterium*, *Niallia circulans*, *Heyndrickxia coagulans*, *Paenibacillus durus*, *Paenibacillus macerans*, *Bacillus subtilis*, and *Bacillus velezensis*, employ multiple mechanisms to promote plant growth. These include nitrogen fixation, phosphorus solubilization, phytohormone production, siderophore synthesis, hydrolytic enzyme secretion, and enhanced tolerance to biotic and abiotic stresses (Goswami et al. 2016; Saxena et al. 2020; Singh et al. 2020). Beyond their growth-promoting capabilities, Bacillaceae members are also renowned for their biocontrol potential, producing a diverse array of antimicrobial metabolites that inhibit bacteria, fungi, insects, nematodes, and viruses (Chowdhury et al. 2015; Miljaković et al. 2020). Notably, cyclic lipopeptides (e.g., iturins, fengycins, and surfactins) and polyketides (e.g., bacillaene, difficidin, and macrolactin) are key compounds responsible for their antifungal and antibacterial activities (Chen et al. 2007; Schneider et al. 2007; Ongena and Jacques 2008; Wu et al. 2015). The synthesis of these metabolites, which can account for up to 10% of the genome in some species, highlights the significant investment Bacillaceae make in biocontrol mechanisms (Stein 2005; Chen et al. 2009). Additionally, these bacteria can induce systemic resistance in plants through the production of volatile organic compounds and other metabolites, further enhancing plant defense mechanisms (Choudhary and Johri 2009).

Recent advancements in DNA sequencing technologies, bioinformatics, and genome analysis tools have revolutionized bacterial genomics, enabling researchers to explore the intricate details of bacterial genomes with unprecedented precision. These developments have not only facilitated the identification of specific genes but also deepened our understanding of their functional relationships. Comparative genomics, in particular, has emerged as a powerful approach to uncovering previously undetected diversity and elucidating the functional capabilities of bacterial species (Torres Manno et al. 2019; Lobanov et al. 2022). Within the Bacillaceae family, genomic studies have reshaped our understanding of their ecological roles and functional diversity. However, despite the hundreds of species within this family, only a subset exhibits PGPR traits (Miljaković et al. 2020). To optimize their application in agriculture, a comprehensive understanding of the functional diversity among Bacillaceae species and the factors influencing their effectiveness in field conditions is essential.

Wheat, a cornerstone of global agriculture, is a high-yielding and adaptable crop that provides essential carbohydrates and proteins for human nutrition (de Sousa et al. 2021). As a major cereal crop, wheat production is critical for addressing global food security and nutritional needs (Subedi et al. 2023). Wheat-based foods, such as bread and noodles, are dietary staples in many regions, contributing significantly to energy, protein, dietary fiber, and essential nutrient intake (Shewry and Hey 2015). Given its importance in temperate zones and its increasing demand in urbanizing and industrializing nations, enhancing wheat productivity and resilience is of paramount importance.

The microbiota associated with wheat, particularly members of the Bacillaceae family, play a crucial role in shaping the plant’s rhizosphere and endophytic communities. Dominating both soil and rhizosphere environments, Bacillaceae can comprise up to 95% of Gram-positive bacterial populations and exhibit endophytic colonization (Miljaković et al. 2020). They are also prevalent in various wheat organs, including seeds, roots, leaves, stems, and kernels, as evidenced by amplicon sequence variant analyses (Kuźniar et al. 2020). This intimate association extends beyond mere presence, as Bacillaceae contribute significantly to wheat biofortification by enhancing nutrient absorption and accumulation in grains. Through mechanisms such as nutrient fixation, solubilization, oxidation, chelation, and regulation—mediated by enzymes, organic acids, biofilms, phenols, and siderophores—Bacillaceae improve nutrient availability and uptake (Liu et al. 2023). Furthermore, they alleviate various stresses in wheat through direct antagonism and indirect regulation, involving the production of enzymes, antibiotics, polysaccharides, and phytohormones (Liu et al. 2023).

This study aims to explore the functional roles of selected Bacillaceae strains in enhancing wheat health and yield. By isolating and characterizing strains from the wheat rhizosphere, we investigate their interactions with the crop and their potential agricultural applications. Through genomic analysis, controlled bioassays, pot experiments, and field trials, we provide a comprehensive evaluation of the mechanisms underlying their contributions to plant growth promotion and stress alleviation. The findings offer valuable insights into optimizing the use of Bacillaceae in sustainable wheat production.

## Materials and methods

### Extraction of rhizospheric soil and spore-forming strains isolation

Rhizospheric soil samples were collected from 4 to 5 wheat plants (aerial parts discarded). After gently shaking roots to remove loosely attached soil, they were transferred using sterile tweezers into a 250-mL Erlenmeyer flask containing 100 mL of sterile water. The suspension was mixed for 10 min at 70 rpm on a zigzag shaker. Following mixing, roots were aseptically removed using sterile tweezers and rinsed with sterile water in the same container. The rhizospheric suspension was then pasteurized (80 °C for 10 min), after which an aliquot was diluted with sterile distilled water and plated on SP-Agar medium (nutrient broth 8 g/L, MgSO_4_·7H_2_O 0.25 g/L, KCl 1 g/L, CaCl_2_ 0.5 mM, MnCl_2_ 0.01 mM, and ferric-iron ammonium citrate 4.4 g/L, agar 1.5%) for spore-forming strain isolation.

### Screening of spore-forming strains for antimicrobial activity using plate assays

The antifungal activity of bacterial isolates was assessed using a radial inhibition assay against *Fusarium graminearum* CCC 140–2010 (CEREMIC collection). Four test isolates were inoculated as single-point colonies at equidistant positions (90° apart, 2.5 cm from the edge) on potato dextrose agar plates (PDA; 4 g/L potato extract, 20 g/L dextrose, and 15 g/L agar). A 5-mm mycelial plug of actively growing *F. graminearum* was placed at the plate center. Plates were incubated at 22 °C for 72 h or until fungal growth reached the edge of the 120-mm-diameter petri dishes. Antifungal activity was quantified by measuring the radius (in mm) of the inhibition zone along the colinear axis between fungal and bacterial colony centers using a manual caliper (Supplementary Fig. 1). While many *Bacillus* strains produce irregular inhibition zones, this standardized approach enabled reliable comparative assessment of antagonistic activity despite morphological variability in inhibition patterns. All assays were performed in triplicate under sterile conditions.

The antibacterial activity of isolates was evaluated against the indicator strain *Pseudomonas syringae* OR1 using a modified overlay inhibition assay. Test strains were inoculated as single-point colonies onto PDA plates and incubated at 30 °C for 72 h to establish growth. Concurrently, the indicator strain was cultured in Luria–Bertani broth (LB; 10 g/L tryptone, 5 g/L yeast extract, and 10 g/L NaCl) at 25 °C with constant agitation (220 rpm) for 16 h. For the overlay, 0.2 mL of the *P. syringae* OR1 culture (1.0 × 10^8^ CFU/mL) was aseptically mixed with 10 mL of molten LB soft agar (0.5% agar) maintained at 45 °C and then immediately poured over the pre-grown test strains on PDA plates. After 48 h of incubation at 25 °C, antibacterial activity was quantified by measuring the radius of the clear inhibition zones surrounding test strains where *P. syringae* OR1 growth was suppressed. Selected strains were deposited in the public culture collection of Acuario del Río Paraná (https://www.santafe.gob.ar/acuario/laboratorio-mixto, Universidad Nacional de Rosario, Santa Fe, Argentina).

### Genomic DNA extraction

Genomic DNA extraction was carried out starting from 10 mL of culture grown in LB broth with permanent shaking at 220 rpm for 16 h. Then, it was centrifuged for 5 min at 6000 rpm and washed with 1 mL of TE buffer (10 mM Tris–HCl and 1 mM EDTA). The cells were resuspended in 2 mL of buffer solution (TE supplemented with RNAse 10 µg/mL) and lysozyme at 5 mg/mL. After a 45-min incubation at 37 °C, proteinase K was added to a final concentration of 1 mg/mL. After 20 min of incubation at 37 °C, 200 µL of 10% SDS was added. A solvent extraction was carried out always preserving the aqueous phase using sequentially 1 volume of phenol equilibrated with TE at pH7, 1 volume of a 1:1 phenol:chloroform mixture and 1 volume of chloroform. Genomic DNA was precipitated by adding sodium acetate at a concentration of 0.3 M and 2.5 volumes of cold ethanol. After centrifuging in a refrigerated centrifuge at maximum speed, the DNA pellet was rinsed with 70% ethanol and allowed to dry at room temperature. Finally, the DNA was resuspended in 300 µL of milliQ water.

DNA quantification was carried out by spectrophotometric measurements in PowerWave TMXS Microplate reader (BioTek, Vermont, USA). For DNA quantification, 196 μL of distilled water was added to 4 μL of sample. Subsequently, the absorbance of the sample was determined at 260 nm, considering that 1 unit of corrected absorbance is equivalent to an amount of DNA of 50 ng/μL. The A260/A280 ratio was also evaluated to determine its purity.

### Hierarchical ordering based on RAPD profiles of isolates

The random amplified polymorphic DNA (RAPD) reactions were performed according to Suarez et al. (2012) in a final volume of 25 μL, using 1 μL of template DNA, 2.5 μM of RAPD19 (5′ GGTCGACYTTNGYN GGRTC 3′) or M13 (5′ GAGGGTGGCGGTTCT 3′) primers, 1 U of Taq Pegasus DNA polymerase (PBL, Buenos Aires, Argentina), 200 μM of each deoxyribonucleotide (dNTP), and 1.5 mM of MgCl_2_, in buffer solution (10 mM Tris–HCl, pH 8, and 50 mM KCl). The amplification reactions were carried out in a TC-3000X Thermal Cycler (Techne, NJ, USA), according to the following protocol: the samples were previously heated to 94 °C for 2 min and, subsequently, were subjected to 40 denaturation cycles (30 s at 93 °C), ringed (90 s at 36 °C) and polymerization (120 s at 72 °C). At the end of the last cycle, a polymerization stage was carried out for 10 min at 72 °C. The corresponding PCR products were separated on an agarose gel 2% buffer TAE 1 ×, stained with SYBER Safe®, and the subsequent analysis was carried out with the free software Gel Analyzer. The presence or absence of a particular PCR product was used as a binary score (present = 1; absent = 0) to perform a hierarchical clustering using “pvclust” R package (Suzuki and Shimodaira 2006), with Manhattan distance and average agglomerative clustering. Clustering reliability was assessed by bootstrapping with 10,000 repetitions.

### Genome sequencing, assembly, and annotation

ZAV-W102, ZAV-W113, ZAV-W127, and ZAV-W35 genomic DNA were prepared for sequencing using the Nextera XT library preparation kit following the manufacturer’s suggested protocols. The prepared libraries were sequenced using a MiSeq DNA sequencer with the MiSeq V3 2 × 300 sequencing kit. The resulting reads were quality trimmed to the Q30 confidence level. The draft genomes were assembled using CLCbio Genomics Workbench 11.0 (Qiagen Inc., Cambridge, MA) using default parameters. ZAV-W70 and ZAV-W64 genomic DNA were sequenced by SNPsaurus using PacBio sequencing technology and assembled with Canu 1.7. Genomic sequences for comparative studies were retrieved from GenBank (ftp.ncbi.nlm.nih.gov/genomes/) using Download Genomes tool (https://github.com/torresmanno/Download_Genomes). Reference strains were defined based on RefSeq or EZBioCloud databases (Yoon et al. 2017). Sequencing and assembly data are available in the NCBI BioProject database under the accession PRJNA1234655. The complete list of genome sequences used in this study is listed in Supplementary Table S1. The genomes were annotated by Prokka software (Seemann 2014) and RAST (Overbeek et al. 2014) using the default settings.

### Species assignment based on genomic data

Average nucleotide identity (*ANI*) values of strains under analysis were calculated with FastANI V1.1 (Jain et al. 2018) program using default parameters. *ANI* threshold value for species circumscription was set at 96%, as previously suggested (Lee et al. 2016; Liu et al. 2017). The maximum likelihood phylogenetic trees were constructed as reported, according to Espariz et al. (2016) with minor modifications. Briefly, the genes present in all analyzed strains (common ancestral genes) were identified by BLASTN (Blast + 2.7.1) searches (Johnson et al. 2008) using an *E*-value of 10^−30^. Common ancestral genes were individually aligned by Clustal Omega V1.2.2 (Sievers et al. 2011) and trimmed using GBlock 0.91b (Talavera and Castresana 2007). Then, aligned genes were concatenated using Python3 package AMAS 0.98 (Borowiec 2016). To remove poor informative sequence regions, those highly similar were not included in the analyses. Finally, phylogenetic relationships of strains were inferred using RAxML 8.2.12 software (Stamatakis 2014) and the GTR substitution model with Gamma distribution. The inferred tree reliabilities were evaluated by bootstrapping with 100 replicates (Stamatakis 2014). The resulting dendrograms were displayed and annotated using iTOL (Letunic and Bork 2011).

### Genomic identification of biocontrol and plant growth promotion-associated genes and clusters

Orthologous proteins were identified using BLASTP searches with an *E*-value threshold of 10^−5^ and a minimum coverage of 50%. For each protein in the model strain, the best hit in the target strains was considered a putative ortholog (based on the highest BLAST score), while additional matches were classified as trivial paralogs. Orthology cut-offs were determined using GeM-Pro software (Torres Manno et al. 2019), which employs trivial paralogs as internal witnesses of non-orthology (IWNO) to refine ortholog identification. For each strain, orthology cut-off values were calculated based on the percentage identity (%ID) of IWNO pairs. If no IWNO pairs were detected for a strain, the mean PID cut-off of its phylogenetic group was applied. Orthologous pairs were verified if their %ID exceeded the corresponding PID cut-off value. Synteny analysis was performed to assess the conservation of gene order, locus completeness, and total nucleotide length of the pathways or loci under study. Biosynthetic gene clusters for secondary metabolites were identified using antiSMASH 5.0 (Blin et al. 2019) with default parameters.

### In vitro determination of plant-growth-promoting properties

The relative indole acetic acid concentration of strain supernatants was determined using the Salkowski method (Goswami et al. 2015) in LB broth supplemented with 25 mM tryptophan (Sigma Aldrich, Saint Louis, MO, USA). To measure the production of volatile compounds (acetoin and 2,3 butanediol) the Voges–Proskauer method was used, as described in Mortera et al. (2013). The analysis of the phosphate solubilization was conducted as indicated in Almirón et al. (2025). The siderophore and biosurfactant productions were assayed in CAS-agar medium, according to Louden et al. (2011), and drop collapse test, as indicated in Tugrul and Cansunar (2005), respectively.

The swarming motility was measured, as described in Niazi et al. (2014). Biofilm formation was analyzed as reported, according to O’Toole and Kolter (1998). Briefly, 200 µL of an overnight (ON) culture diluted to OD 0.08 was placed in multi-well plates, incubated for 6 h at 30 °C, and the supernatant was discarded and plates were on drought for 30 min at 42 °C. Then, the plates were washed with distilled water to remove non-adherent cells, and 300 μL of 1% w/v Crystal Violet was added to each well and incubated for 15 min at room temperature. After removing the excess dye by washing the plates with distilled water, 200 mL of 95% v/v ethanol was added to each well. Adhesion was quantified by determining the absorbance at 540 nm. The exoenzyme activities of protease, lipase, lecithinase, esterase, and amylase were determined according to Ficarra et al. (2016); cellulase to Ariffin et al. (2006); xylanase to Amore et al. (2015); chitinase to Rodriguez-Kabana et al. (1983). All the experiments were repeated at least by triplicate, and similar results and a representative experimental result are shown.

### Analysis of the biocontrol in wheat seeds

The antifungal activity of bacterial strains was evaluated using bacterial-free supernatants against *F. graminearum* in sealed glass flasks. The cultures of bacterial strains were grown ON in PD broth at 28 °C under constant agitation (150 rpm). Then, bacterial-free supernatants were obtained by centrifuging ON cultures at 10,000 rpm for 30 min and filtering the supernatant through a sterile 22-µm syringe filter (Nalgene). In sterile 100-mL glass flasks, 15 g of healthy, mature, and homogeneous wheat seeds previously disinfected (the seeds were submerged in 70% ethanol during 5 min, and then rinsed three times with sterile distilled water, and finally the water is discarded and kept dry) were mixed with 4 mL of bacterial supernatant, sterile distilled water (negative fungicide control), PD broth (negative fungicide control), or Metalaxyl® solution (10 µg/mL, positive fungicide control). The flasks were manually agitated to ensure uniform seed coverage and then challenged with *F. graminearum* by adding 500 µL of a spore suspension (1.0 × 10^5^ spores/mL; spores were counted in Neubauer counting chamber). Control flasks without fungal infection received an equivalent volume of sterile distilled water. The flasks were incubated in a humidity chamber at 27 °C, and infection symptoms were monitored at 3 to 5 days post-inoculation. Each treatment was performed in triplicate, and assays were conducted in duplicate. At 5 days post-infection, images of the flasks were analyzed using ImageJ (Schneider et al. 2012) to quantify the extent of infection, expressed as the percentage of white area (mycelial growth) relative to the total seed surface.

### Growth promotion assays on wheat seedlings under controlled conditions

To evaluate the growth-promoting effects of bacterial strains on wheat seedlings, experiments were conducted under controlled environmental conditions. The bacterial inoculant suspensions were obtained by ON cultures of the strains grown in PD broth at 28 °C under constant agitation (220 rpm). The cultures were diluted with sterile distilled water to a bacterial concentration of 1 × 10⁹ CFU/mL. Healthy, mature, and homogeneous wheat seeds were selected and immersed by using a tweezer in the bacterial inoculant suspensions for 5 min under constant agitation. Control treatments consisted of PD broth diluted in sterile distilled water at the same ratio used for bacterial cultures. Treated seeds were sown in plastic multi-pot trays containing a 1:1 mixture of sterile sand and perlite as the substrate. Two seeds were planted per compartment, with a total of 12 plants per treatment. The trays were irrigated with demineralized water and placed in a greenhouse with a 14/10 h light/dark photoperiod (150 µE m⁻2 s⁻1), controlled temperature (25–27 °C), and relative humidity (50–60%). To prevent cross-contamination between strains, a separate multi-pot tray was used for each treatment (strains and control), and trays were rotated regularly to ensure uniform growth conditions.

At 15 days after sowing, plant growth parameters were measured. The greenness index, an indicator of chlorophyll content and nutritional status, was determined using a SPAD 502 Plus (Minolta Co.). The length of the first and second leaves was measured, and the total leaf area was calculated using the allometric index described by Chanda and Singh (2002). Seedlings were then harvested, carefully cleaned, and separated into aerial parts and roots. Fresh weights of both parts were recorded. Root morphology, including total length, average diameter, volume, surface area, and number of tips, was analyzed using a root scanner and WinRhizo® software (Bouma et al. 2000). Finally, both aerial parts and roots were dried at 60 °C until constant weight to determine dry weights. Each strain was tested in three independent experiments under identical conditions.

### Field trials assessing bacterial effects on wheat growth and yield

Field experiments were conducted over two growing seasons (2022 and 2023) to evaluate the effects of bacterial strains and fungicide treatments on wheat yield. Six treatments were established: T1 (water control), T2 (ZAV-W64 strain), T3 (ZAV-W70 strain), T4 (fungicide, Metalaxyl®), T5 (fungicide + ZAV-W64 strain), and T6 (fungicide + ZAV-W70 strain). The ZAV-W64 and ZAV-W70 strains were grown in PD broth, as previously described and formulated to a concentration of 10⁸ CFU/mL (Navira SA). Wheat seeds were treated according to standard agronomic practices. For bacterial treatments, 20 mL of inoculant per kg of seeds was applied by manual shaking in a plastic bag. For fungicide treatments, 2 mL of Metalaxyl® solution (10 µg mL⁻1) per kg of seeds was used. Combined treatments (T5 and T6) included both the fungicide and bacterial inoculant at the same concentrations.

The 2022 field trial was conducted at the Unidad Experimental de Cultivos Extensivos (UECE) of the Facultad de Ciencias Agrarias (UNL) in Esperanza, Santa Fe (− 31° 24′ 56″ S, 60° 54′ 28″ W), on a Typical Argiudol soil from the Esperanza series. Field plots, measuring 3 m wide by 10 m long, were sown using an experimental seeder with a row spacing of 17.5 cm. The short-cycle wheat variety Nutria (Klein seedbed) was sown on July 12, 2022. The crop was maintained free of pests (insects and weeds) and diseases. At physiological maturity, plants from the central rows covering three equally distributed 1 m^2^ areas were manually harvested and threshed using an experimental thresher. The following variables were recorded: yield (*Y*, in kg ha⁻^1^), number of spikes per square meter (SN m⁻^2^), weight of 1000 grains (P1000), and biomass dry weight.

The 2023 field trial was conducted at the Estación Experimental del INTA in Oliveros, Santa Fe (− 32° 34′ 26″ S, 60° 52′ 15″ W), on a Typical Argiudol soil from the Maciel series. Four field plots per treatment were arranged in a complete block design, each measuring 3 m wide by 12 m long. The intermediate-cycle wheat variety Buck SY-109 (Buck seedbed) was sown on June 22, 2023, using an experimental seeder with a row spacing of 20 cm. The crop was maintained free of pests (insects and weeds) and diseases. Prior to harvest, the number of plants per m^2^ was recorded in a representative section of each plot. At physiological maturity, the entire plots were harvested using an experimental harvester, and the following variables were determined: yield (*Y*, in kg ha⁻^1^),number of spikes per square meter (SN m^⁻2^), weight of 1000 grains (P1000), and plant height (as a biomass predictor). In order to predict bread wheat grain quality, six major parameters were measured: total protein percentage, wet gluten percentage, alveograph W parameter, test weight, ash, and moisture. A total of 200 g of wheat kernels were analyzed by near infrared NIRS™ DS2500 L (FOSS Analitycs), and values were estimated by regression curves of INTA data base.

### Statistical analysis

Statistical analyses were performed using InfoStat software, version 2020 (Di Rienzo et al. 2020). The effects of treatments on kernel antifungal capacity were evaluated using a unifactorial ANOVA (treatment factor), with mean comparisons conducted using the LSD test. For seedling growth promotion assays, a two-factor mixed model ANOVA (treatment and assay factors) was applied, followed by the LSD multiple comparison test. Field assay data were analyzed using a two-factor mixed model ANOVA (treatment and fungicide factors), with mean comparisons made using the LSD test. The assumptions of ANOVA, including normal distribution of residuals (assessed using the Shapiro–Wilk test and QQ plots) and homoscedasticity (evaluated using the Levene test and residual plots), were verified for all analyses. For variables that did not meet ANOVA assumptions (e.g., in seedling growth promotion trials), non-parametric Kruskal–Wallis tests were performed, with data partitioned by assay.

## Results

### Isolation of Bacillaceae strains from wheat rhizosphere and screening for antimicrobial activity and genetic diversity

To harness the native potential of Bacillaceae associated with wheat, particularly their adaptability to rhizospheric conditions, we implemented a targeted isolation strategy focused on spore-forming microorganisms, a key characteristic of the family. Rhizospheric soil samples from wheat cultivations were heat-treated and plated on solid media. A total of 576 colonies displaying flat surfaces, no pigmentation, and speculated, filamentous, or wavy edges were selected. Gram staining confirmed their Gram-positive nature.

The antagonistic activity of these isolates was evaluated against the global pathogen *F. graminearum*, which significantly impacts grain quality and yield through mycotoxin production (Liu et al. 2023). Among the 576 isolates, 131 exhibited antifungal activity against *F. graminearum* CCC 140–2010, with inhibition halos ranging from 1 to 9 mm (Supplementary Table S2). Additionally, 65 isolates showed antagonistic activity against *P. syringae* OR1, with inhibition halo radii ranging from 1 to 6 mm (Supplementary Table S2).

To ensure robust strain differentiation, we employed RAPD analysis, which revealed 39 distinct polymorphic patterns (Supplementary Table S2). A dendrogram based on RAPD clustering is provided in Supplementary Figure S2, illustrating the genetic diversity among the isolates. From the 131 isolates exhibiting antifungal activity, a subgroup of 28 isolates, distinguished by differences in colony morphology, inhibition halo radius, and RAPD clustering, was selected for 16S rRNA analysis. This analysis confirmed their taxonomic affiliation, with 2, 13, and 13 isolates belonging to *B. amyloliquefaciens*, *B. cereus*, and *P. megaterium*, respectively, with 99–100% sequence identity (Supplementary Table S2). Notably, *B. cereus* isolates were excluded from further study due to their potential association with human pathogenicity (Torres Manno et al. 2020). Finally, six representative isolates with diverse morphological, taxonomic, and genotypic characteristics, were selected based on their superior antimicrobial profiles. These strains, listed in Table 1, are chosen for further investigation to explore their potential in biocontrol, plant growth promotion, and yield enhancement in wheat.

**Table 1:** Antimicrobial characteristics and species assignations of the selected isolates

Strain	RAPD polymorphic cluster	Antifungal activity (mm)*	Antibacterial activity (mm)*	ANI values with type strain of the assigned species**	Species assignation
ZAV-W70	11	6.7 ± 0.6	ND***	97.9%	*B. velezensis*
ZAV-W102	21	5.3 ± 1.5	7.0 ± 1.0	98.1%	*B. velezensis*
ZAV-W35	17	1.3 ± 0.6	5.7 ± 3.2	96.0%	*P. megaterium*
ZAV-W64	3	2.0 ± 1.0	6.7 ± 2.1	96.0%	*P. megaterium*
ZAV-W113	4	2.0 ± 1.0	2.3 ± 1.5	96.1%	*P. megaterium*
ZAV-W127	1	3.0 ± 1.0	5.3 ± 3.5	96.1%	*P. megaterium*

^*^ The antifungal activity was determined against *F. graminaerum* CCC 140–2010 and the antibacterial activity against *P. syringae* OR1. The means ± standard deviations of the experiments performed in triplicate are shown. ** Type strains *P. megaterium* ATCC 14581^ T^ or *B. velezensis* NRRL B-41580^ T^ were used for ANI calculations

^***^ND, not determined. The rapid growth of the ZAV-W70 strain prevented the plaque assay

### Species-level identification through whole-genome analysis

To elucidate the role of distinct Bacillaceae species within the wheat rhizosphere, unequivocal species-level identification is essential, particularly given the ongoing reclassification within this taxonomic family. While 16S ribosomal gene analysis initially grouped our selected isolates as *P. megaterium* or *B. amyloliquefaciens*, it lacked the resolution for precise species-level identification. Therefore, we employed a phylogenomic approach, constructing phylogenetic trees based on selected core genes from the genomic sequences of the isolates and type strains of *P. megaterium* or *B. amyloliquefaciens* taxonomic groups.

Notably, strains ZAV-W127, ZAV-W113, ZAV-W35, and ZAV-W64 clustered within the branch of the type strain *P. megaterium* ATCC 14581^ T^ (Supplementary Figure S3A). In contrast, strains ZAV-W70 and ZAV-W102 grouped with the type strain *B. velezensis* NRRL B-41580^ T^ within the *B. amyloliquefaciens* operational group (Supplementary Figure S3B). These species assignations were further supported by *ANI* values exceeding 96% with their phylogenetically closest type strains (Table 1).

This integrated phylogenomic and ANI-based approach ensures robust species-level identification of our selected wheat rhizosphere isolates, providing a solid foundation for subsequent in-depth analyses of their functional roles and potential contributions to plant–microbe interactions.

### Genomic mining of biocontrol and plant growth promotion pathways in selected Bacillaceae strains

To gain deeper insight into the genetic potential of the selected strains, we conducted a comprehensive whole-genome analysis using comparative genomics. Understanding the biocontrol and plant growth promotion mechanisms of Bacillaceae is crucial for the targeted selection of strains suitable for agricultural applications (Miljaković et al. 2020). Here, we employed the GeM-Pro algorithm (Torres Manno et al. 2019) to explore the genetic basis of these traits in the selected isolates, using known biocontrol and plant growth promotion genes as queries.

Notably, the *B. velezensis* isolates (ZAV-W70 and ZAV-W102), which exhibited superior antifungal activities (Table 1), displayed a higher abundance of pathways involved in the production of fungicides, including fengycins, and Iturin A (Table 2 and Supplementary Table S3) (Dunlap et al. 2019). Additionally, these strains harbored genes related to the biosynthesis of antibacterial metabolites such as bacillaene, surfactin, difficidin, bacillysin, and amylocyclicin (Scholz et al. 2014; Mora et al. 2015). Furthermore, they encoded pathways for the degradation of xylan and cellulose, highlighting their potential for enhancing nutrient availability in the rhizosphere.

**Table 2:** Pathways associated to biocontrol and/or plant growth promotion detected by in silico whole-genome mining

Pathway	*B. velezensis*		*P. megaterium*			
	ZAV-W70	ZAV-W102	ZAV-W35	ZAV-W64	ZAV-W113	ZAV-W127
Associated with antifungal activities						
Iturin A	Present	Present	NF	NF	NF	NF
Fengycin	Present	Present	NF	NF	NF	NF
Associated with antibacterial activities						
Bacillaene	Present	Present	NF	NF	NF	NF
Difficidin	Present	Present	NF	NF	NF	NF
Macrolactin	NF	NF	NF	NF	NF	NF
Surfactin	Present	Present	NF	NF	NF	NF
Bacillysin	Present	Present	NF	NF	NF	NF
Amylocyclicin	Present	Present	NF	NF	NF	NF
Plantazolicin	NF	NF	NF	NF	NF	NF
Associated with plant growth promotion						
IAA	Present	Present	Present	Present	Present	Present
Acetoin	Present	Present	NF	NF	NF	NF
H2S	Present	Present	Present	Present	Present	Present
GABA	Present	Present	Present	Present	Present	Present
PAA	NF	NF	Present	Present	Present	Present
Bacillibactin	Present	Present	NF	NF	NF	NF
Lactonase	NF	NF	NF	NF	NF	NF
Petrobactin	NF	NF	NF	NF	NF	NF
Polyamine	Present	Present	NF	NF	NF	NF
Exoenzyme production						
Amylase	Present	Present	Present	Present	Present	Present
Cellulase	Present	Present	NF	NF	NF	NF
Xylanase	Present	Present	NF	NF	NF	NF
Chitinase	NF	NF	NF	NF	NF	NF
Associated with other phenotypes						
Biofilm	Present	Present	NF	NF	NF	NF

The presence of genes involved in biocontrol and/or plant growth promotion was searched using BLAST; false positive orthologous genes were filtered by using cut-offs defined using the “Internal-witness of non-orthology” criterion and synteny analysis described in Torres Manno et al. (Torres Manno et al. 2019). See Supplementary Table S3 for the complete list of genes involved in each analyzed pathway

*NF* not found or identified (including false positives)

To further elucidate the functional potential of ZAV-W70 and ZAV-W102, we conducted a comparative genomic analysis against genomes of *B. velezensis, B. amyloliquefaciens*, and *B. siamensis* that belong to the same taxonomic operational group (*B. amyloliquefaciens*) (Fan et al. 2017)*.* First, all strains were rigorously validated through ANI analysis (> 96% identity with type strains) and phylogenetic placement (Supplementary Figure S3A), ensuring accurate taxonomic classification prior to comparative analysis. Among *B. velezensis* strains, we observed high conservation of gene clusters for macrolactin (present in 97% of strains), difficidin (92%), and fengycin (79%), compared to < 29% prevalence in the other species. Notably, plantazolicin showed restricted distribution, being present in only 15% of *B. velezensis* genomes and completely absent from *B. amyloliquefaciens* and *B. siamensis*. Strains ZAV-W102 and ZAV-W70 exhibited a rare genomic profile (shared by only 2% of *B. amyloliquefaciens* operational group strains) characterized by the presence of fengycin but absence of macrolactin clusters. These findings highlight the unique genetic potential of *B. velezensis* strains for antimicrobial activity and plant growth promotion.

The *P. megaterium* strains (ZAV-W35, ZAV-W64, ZAV-W113, and ZAV-W127) displayed genomic signatures for volatile organic compound production and plant growth-promoting metabolites, including phytohormones (e.g., IAA) and siderophores (Table 2 and Supplementary Table S3). Following taxonomic validation through ANI analysis (> 96% identity with type strains) and phylogenetic placement (Supplementary Figure S3B), comparative genomic analysis of 126 *Priestia* genomes (encompassing *P. megaterium*, *P. aryabhattai*, and *P. flexus*) revealed both conserved and variable traits across the genus. While biosynthesis pathways for H₂S, AIA, polyamines, and GABA were universally present, the phenylacetic acid (PAA) pathway — a known inhibitor of *Fusarium oxysporum* spore germination (Wu et al. 2020) — showed distinct species-specific patterns, occurring in only 11% of *P. megaterium,* 14% of *P. aryabhattai*, and 60% of *P. flexus* genomes. Notably, ZAV-W35, ZAV-W64, ZAV-W113, and ZAV-W127possessed the PAA pathway genes, relatively uncommon for *P. megaterium* strains.

### Analysis of phenotypic traits associated with plant growth promotion

The in silico predictions regarding the isolates’ capacity to produce amylase, xylanase, IAA, and biosurfactants were experimentally validated, confirming their functional relevance (Table 3). Notably, despite the absence of well-known siderophore gene clusters, such as bacillibactin (Chen et al. 2009), in the *P. megaterium* genomes, our siderophore production assays revealed iron-chelating capabilities in all strains. To explore this further, we used the antiSMASH framework to detect clusters of co-occurring biosynthesis genes, identifying a region with 62% similarity to the siderophore schizokinen synthesis cluster (Table 3).

**Table 3:** Activities of the selected isolates associated to biocontrol and/or plant growth promotion detected by in vitro assays

Test	*B. velezensis*		*P. megaterium*			
	ZAV-W70	ZAV-W102	ZAV-W35	ZAV-W64	ZAV-W113	ZAV-W127
Biofilm formation*	1.89 ± 1.75	1.20 ± 0.97	0.20 ± 0.05	0.243 ± 0.10	0.12 ± 0.05	0.14 ± 0.05
Swarming mobility	+ +	+ +	(−)	+	(−)	(−)
IAA**	0.62 ± 0.47	0.21 ± 0.11	0.06 ± 0.02	0.04 ± 0.04	0.05 ± 0.06	0.03 ± 0.04
Acetoin production	+ + +	+ +	(−)	(−)	(−)	(−)
Siderophores	+ + +	+ + +	+	+	+	+
Biosurfactant	+ + +	+ + +	−	−	−	−
Amylase*	3.50 ± 0.50	4.00 ± 1.32	0.67 ± 0.29	1.83 ± 0.29	1.67 ± 0.58	1.33 ± 0.58
Cellulase*	7.10 ± 0.76	4.00 ± 1.00	0 ± 0	0 ± 0	0 ± 0	0 ± 0
Xylanase	+	+	N/D	N/D	N/D	N/D
Chitinase	(−)	(−)	(−)	(−)	(−)	(−)
Lecithinase	(−)	(−)	(−)	(−)	(−)	(−)
Lipase	+	+	(−)	(−)	(−)	(−)
Esterase	+	+	(−)	(−)	(−)	(−)
Protease*	5.33 ± 1.04	7.67 ± 0.58	2.67 ± 0.58	3.67 ± 0.29	3.17 ± 0.29	2.67 ±.029
Phosphate solubilization	+ +	+ +	+	+	+	+

Qualitative results are indicated as + for positive (+ +  + high, +  + medium o + low intensity) or (−) for negative results

*N*/*D* not determined activities

^*^ Radius measurements in mm; ** Absorbance values

The *B. velezensis* isolates (ZAV-W70 and ZAV-W102) exhibited the highest proteolytic activities, along with previously noted amylolytic and cellulolytic capabilities. Additionally, they uniquely demonstrated the ability to produce lipases and esterases, distinguishing them from the *P. megaterium* strains (Table 3). Both *B. velezensis* and *P. megaterium* isolates showed the ability to solubilize phosphate and form biofilms, although biofilm formation was more pronounced in *B. velezensis* (Table 3).

The integration of in silico predictions and experimental validations underscores the complementary value of these approaches, enhancing our understanding of the phenotypic traits associated with plant growth promotion in the selected Bacillaceae strains.

### Evaluation of biocontrol potential against fungal contaminants in wheat seeds

**Fig. 1:**
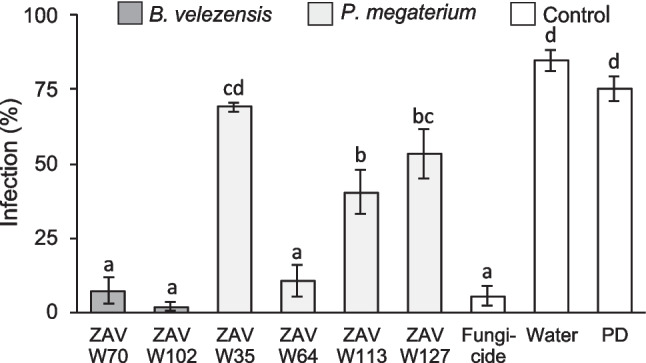
Antifungal activity of selected Bacillaceae strains on wheat seeds. Quantification of antifungal activity using bacterial-free supernatants. Wheat seeds were treated with supernatants, sterile distilled water and PD broth (negative fungicide control), or Metalaxyl® solution (positive fungicide control), followed by inoculation with *F. graminearum* (1.0 × 105 spores/mL). Infection symptoms were quantified at 5 days post-inoculation using ImageJ, expressed as the percentage of mycelial growth relative to the total seed surface. Values are *means* ± *SEM* (*n* = 3). Bars not sharing the same letter are significantly different according to ANOVA and LSD multiple comparison test (*p* < 0.05)

To determine whether antifungal activity was mediated by secreted metabolites, we performed a kernel bioassay using cell-free supernatants to inhibit *F. graminearum* growth on sterilized wheat seeds. The *B. velezensis* strains ZAV-W70 and ZAV-W102 effectively prevented seed infection, exhibiting similar efficacy to the chemical fungicide used as a positive control, showing average infections values lower than 10% (Fig. 1 and Supplementary Figure S4). Among the *P. megaterium* strains, ZAV-W64 demonstrated significantly higher inhibitory activity, compared to the other isolates of the specie (infection average of 10%), although all strains showed some level of antifungal activity (Fig. 1 and Supplementary Figure S4). The negative controls of antifungal activities were significantly higher than the other treatments (infection average upper than 80%), in which water or PD broth presented similar values (Fig. 1 and Supplementary Figure S4). When the inoculum of *F. graminearum* was replaced with water (negative control of infection), no fungal growth was observed as expected (data not shown).

These findings underscore the potential of *B. velezensis* ZAV-W70 and ZAV-W102, as well as *P. megaterium* ZAV-W64, as promising candidates for biocontrol applications in wheat. Their ability to inhibit *F. graminearum* highlights their versatility and effectiveness in protecting wheat seeds from fungal pathogens.

### Evaluation of plant growth properties in seedlings assays growth under controlled conditions

**Fig. 2:**
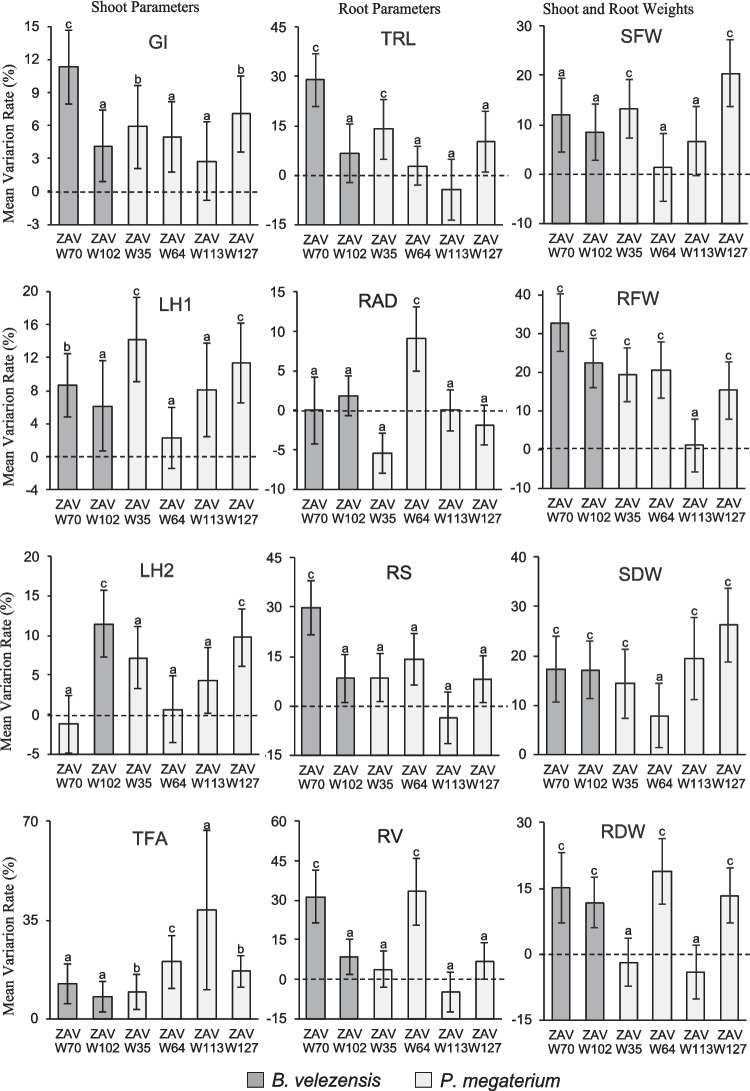
Plant growth promotion of selected Bacillaceae strains in seedlings assays growth under controlled conditions. The figure summarizes the results obtained in trials of seedlings growth under controlled conditions across three repetitions. Values are expressed as *means* ± *SEM* (*n* = 36). Values not sharing any letter are significantly different according to ANOVA LSD multiple comparison test (*p* < 0.05). Measured variables included shoot parameters (greenness index = *GI*, the length of the first leaf = LH1, and the second leaf = LH2, total foliar area = TFA), root parameters (total length = *RTL*; average diameter = *RAD*; surface = *RS*; volume = *RV*), and seedling weights (shoot fresh weight = *RFW*, root fresh weight = *SFW*, shoot dry weight = *SDW*, root dry weight = *RDW*). The root tip number (*RNT*) parameter was excluded, as it showed no significant differences. In the statistical notation, the letter c indicates a significant increase, compared to the control across all three replicates (p < 0.01); b denotes a significant increase in one replicate (*p* < 0.01), while a shows no significant difference from the control in any replicate. The parameters further describe in the results section were those with significant differences in the three experimental replicates

To assess the ability of the selected isolates to enhance plant growth, we conducted pot assays with wheat seeds under controlled conditions. All tested *B. velezensis* and *P. megaterium* strains significantly improved several plant growth parameters compared to controls, though the patterns of improvement varied between strains, even within the same species (Fig. 2 and Supplementary Table S4).

The *B. velezensis* strains, ZAV-W70 and ZAV-W102, exhibited notable improvements in structural parameters, including shoot and root dry weights, as well as the physiological parameter root fresh weight (Fig. 2 and Supplementary Table S4). Specifically, ZAV-W70 demonstrated significant increases in three root-related measures (length, surface area, and volume) and the greenness index compared to the control (Fig. 2 and Supplementary Table S4).

Among the *P. megaterium* strains, ZAV-W35, ZAV-W64, ZAV-W113, and ZAV-W127 showed significant improvements in four, five, one, and six growth parameters, respectively (Fig. 2 and Supplementary Table S4). Notably, ZAV-W64 and ZAV-W127 both increased root fresh and dry weights, but only ZAV-W64 significantly enhanced root diameter and volume (Fig. 2 and Supplementary Table S4). While ZAV-W127 improved leaf length and shoot fresh and dry weights, these changes were not reflected in total leaf area, unlike ZAV-W64, which showed a significant increase in this parameter (Fig. 2 and Supplementary Table S4).

These results confirm the association between the previously described genetic and phenotypic traits (Tables 2 and 3) and the observed plant growth promotion capabilities. The *B. velezensis* strain ZAV-W70 and the *P. megaterium* strain ZAV-W64 emerged as particularly promising candidates for enhancing plant growth, demonstrating consistent and significant improvements across multiple growth parameters.

### Impact of *B. velezensis* ZAV-W70 and *P. megaterium* ZAV-W64 on wheat yield and seed quality in field trials

**Fig. 3:**
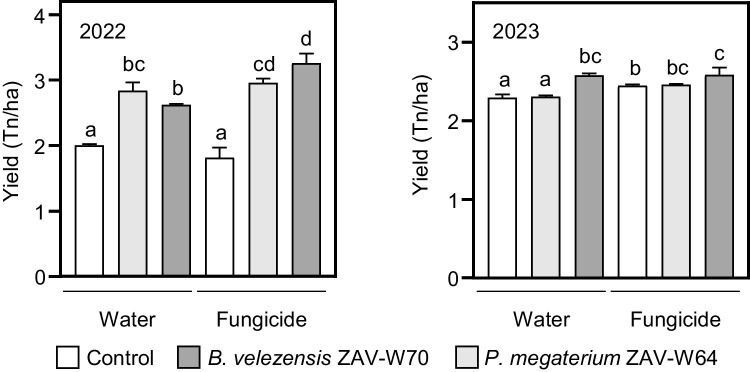
Effects of *P. megaterium* ZAV-W64 and *B. velezensis* ZAV-W70 on wheat yield under field conditions. Yield results from the 2022 (left panel) and 2023 (right panel) growing seasons. Wheat seeds were treated with ZAV-W64, ZAV-W70, or controls (water and fungicide). Yield values are expressed as *means* ± *SEM* (*n* = 3 for 2022; *n* = 4 for 2023). Bars not sharing any letter are significantly different according to ANOVA and LSD multiple comparison test (*p* < 0.05)

To evaluate the capacity of the selected isolates to enhance wheat productivity, field assays were conducted over two consecutive growing seasons. Based on previous results, we focused on *B. velezensis* ZAV-W70 and *P. megaterium* ZAV-W64 as the most promising strains. In the 2022 season, both strains significantly increased yield, compared to the control in the absence of fungicide (31% for ZAV-W64 and 42% for ZAV-W70) and in combination with fungicide (62% for ZAV-W64 and 79% for ZAV-W70), with no significant differences between the strains (Fig. 3). The overall average yield was 2581.4 kg ha⁻1, with maximum yields of 3261.4 kg ha⁻1 (fungicide and ZAV-W70 combination) and minimum yields of 1814.9 kg ha⁻1 (fungicide alone) (Fig. 3). The fungicide effect was only significant in ZAV-W70 treatments, suggesting an additive effect rather than solely antifungal activity (Fig. 3). Yield component analysis revealed higher biomass in ZAV-W70 treatments with fungicide and an increased number of spikes in ZAV-W64 and ZAV-W70 treatments without fungicide, correlating with yield improvements (Supplementary Table S5A). Additionally, the weight of 1000 grains increased in fungicide treatments, while ZAV-W70 inoculations showed a higher inferred number of grains (Supplementary Table S5A).

In the 2023 season, ZAV-W70 significantly increased yield by 11.7% without fungicide and 5.6% with fungicide, compared to water or fungicide controls, respectively (Fig. 3). ZAV-W64 with fungicide also increased yield by 7%, compared to the water control (Fig. 3). The overall average yield was 2446.1 kg ha⁻1, with maximum yields of 2584.3 kg ha⁻1 (fungicide and ZAV-W70 combination) and minimum yields of 2298.3 kg ha⁻1 (water control) (Fig. 3). The fungicide effect was significant in the control and ZAV-W64 treatments but not in ZAV-W70, indicating that ZAV-W70 alone had a similar effect to ZAV-W70 combined with fungicide, acting as both a growth promoter and a fungicide (Fig. 3). ZAV-W70 treatments led to increased plant height (a proxy for biomass) and a higher number of fertile spikes in treatments without fungicide (Supplementary Table S5B). In contrast, the yield improvement in ZAV-W64 treatments with fungicide was associated with a slight increase in the number of fertile spikes, while grain weight remained similar to the control (Supplementary Table S5B).

**Fig. 4:**
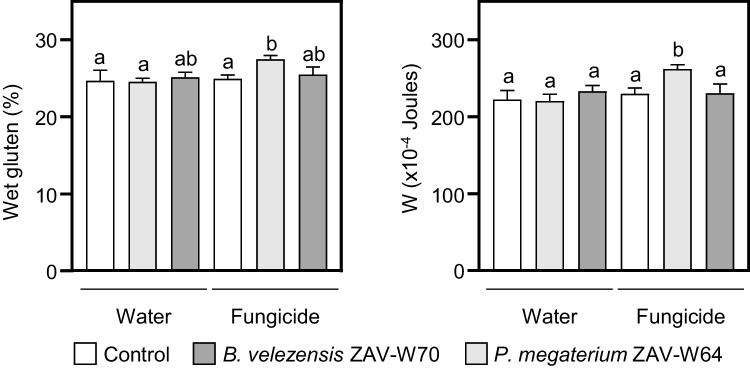
Effects of *P. megaterium* ZAV-W64 and *B. velezensis* ZAV-W70 on wheat seed quality parameters. Wet gluten content (left panel) and alveograph W parameter (right panel), indicating dough strength, in seeds produced from plants grown from seeds treated with ZAV-W64, ZAV-W70, or controls (water and fungicide) prior to sowing. Values are expressed as *means* ± *SEM* (*n* = 3). Bars not sharing any letter are significantly different according to ANOVA and LSD multiple comparison test (*p* < 0.05)

Given the importance of wheat in the human diet, we also evaluated bread-making quality. Seeds treated with ZAV-W64 in combination with fungicide exhibited improved bread-making quality, with significant increases in total gluten (10%) and the alveograph W parameter (15%), compared to fungicide alone (Fig. 4). ZAV-W70 also showed a non-significant increase in gluten percentage, compared to controls (Fig. 4). The increase in total gluten for ZAV-W64 with fungicide correlated with higher protein content (Supplementary Table S5C). Moisture levels were similar across treatments, while water and fungicide treatments showed increases in ash content and test weight, respectively (Supplementary Table S5C).

## Discussion

Soil overexploitation and the extensive use of agrochemicals have drastically reduced microbial diversity and impaired the functional potential of soil microbiota, with significant implications for agricultural productivity (Meena et al. 2020; Gupta et al. 2022). Regenerative agriculture has emerged as a sustainable alternative, promoting environmentally friendly practices aimed at maintaining or restoring soil health over the medium and long term. A key aspect of this approach involves the use of microbial-based bioinputs to reduce or replace agrochemical inputs, targeting both nutrient management and pathogen control (Maitra et al. 2021). This growing interest is reflected in the global agricultural biological market, which was valued at $14.6 billion in 2023 and is projected to reach $27.9 billion by 2028 (Frederick and Mei 2024).

The substantial diversity of the Bacillaceae family necessitated a rigorous selection process to prioritize candidates, constrained by budgetary considerations — a critical factor in any R&D effort. Rapid and straightforward screening methods are, therefore, essential for narrowing down the pool of candidates. Recently, a basic pipeline for the screen, test, validation, application, and evaluation (STVAE) of *Bacillus* spp. used for sustainable wheat production has been proposed (Liu et al. 2023). The selection and testing steps were extensively addressed by in vitro and ex vivo assays (Liu et al. 2023). In this study, we highlighted the pivotal role of genomics in selecting new or improved microbial products tailored to specific crops and environments. The focus was on isolating Bacillaceae strains from the wheat rhizosphere due to their established association with wheat and their documented roles in plant growth promotion and biocontrol (Liu et al. 2023).

Considering the resilient nature of spore-forming bacteria, their isolation from a specific environment does not necessarily imply adaptation to that environment, as their resistance allows them to remain viable under diverse conditions. Therefore, we assessed in vitro antifungal activity against *F. graminearum* as a key phenotype indicative of soil adaptation (Supronienė et al. 2023). Then, we employed the RAPD technique, which complements phenotypic screening by providing a cost-effective method to differentiate genetic profiles and identify clonally distinct strains. RAPD proved valuable in reducing the number of candidates by excluding redundant sequences, ensuring a more streamlined and efficient selection process for downstream genomic analysis. The number of observed distinct polymorphic patterns was notably lower than the expected diversity for the genus, suggesting that some clonally unrelated strains shared indistinguishable profiles (Fig. S1). Nevertheless, this approach allowed us to reduce the number of strains for 16S rRNA sequencing, which, despite its limitations in precise species assignation (Espariz et al. 2016; Chun et al. 2018), enabled the selection of strains belonging to groups reported as growth promoters for whole-genome sequencing. Whole-genome information significantly enhances the candidate selection process in two critical ways. First, it enables precise species identification, allowing for the exclusion of isolates associated with species that have a history of pathogenicity (EFSA Panel on Biological Hazards (BIOHAZ) 2016). Second, genomic analysis facilitates the targeted identification of traits of interest. By employing GeM-Pro (Torres Manno et al. 2019) as an exploratory tool, we conducted a comprehensive characterization of strains, focusing on genes associated with desired functionalities.

Our findings underscore both the potential and limitations of genomic analysis. While predicted genes may not always translate into observable phenotypic activity—possibly due to suboptimal experimental conditions—phenotypic assays may uncover activities not predicted in silico, often due to incomplete or biased databases. For instance, alpha-amylase activity, initially predicted only for *B. velezensis*, was observed in all isolates (Table 3). Database refinement improved predictive accuracy, subsequently identifying amylase-related genes in *P. megaterium* strains (Table 2). However, database limitations remain pronounced for traits governed by complex genetic and regulatory mechanisms. For example, while biofilm formation in *B. velezensis* correlated with genes like *tapA*, *pgcA*, *gtaB*, *epsGE*, and *sipW*, additional genes likely contributed to this phenotype in *P. megaterium*.

The comparative genomic analyses conducted in this study provided valuable insights into the functional diversity of the selected Bacillaceae strains. By comparing the genomes of *P. megaterium* and *B. velezensis* strains with those of closely related species, we identified key metabolic pathways and antimicrobial gene clusters that contribute to their plant growth-promoting and biocontrol capabilities. These analyses revealed that *B. velezensis* strains possess a broader suite of antimicrobial genes, such as those encoding difficidin and fengycin, which are less prevalent in other species like *B. amyloliquefaciens* and *B. siamensis*. Conversely, *P. megaterium* strains exhibited unique traits, such as the production of phenylacetic acid (PAA).

These genomic predictions were subsequently validated through experimental assays, and additional phenotypic traits were assessed to confirm their potential. Likewise, it is expected that rhizospheric bacteria encode for a diverse array of characteristics related to their potential as biocontrol agents or plant promotion (Abedinzadeh et al. 2019). Here, these effects were observed in *B. velezensis* and *P. megaterium* strains, spanning the majority of activities assayed, and confirming the strain selection carried out (Table 3). Noticeably, one activity used as criterion to select isolates during the screening, such as phosphate solubilization (Da Costa et al. 2014; Mukhtar et al. 2017), was observed in *B. velezensis* and more slightly in *P. megaterium* strains.

Even when genomics and in vitro results predicted a high probability of antifungal capacity, determining in vivo biocontrol efficacy remains an inherent challenge (Akinrinlola et al. 2018). The biocontrol using bacterial suspensions was confirmed against naturally occurring storage fungi and applied *F. graminearum*. The *B. velezensis* inhibition by secondary metabolites was previously associated with the conserved fungicides iturin and fengycin (Palazzini et al. 2016). The capacity of a *P. megaterium* strain to inhibit the development of fungi is scarcely reported, just mentioned as possible biocontrol agent on *Aspergillus flavus* (Mannaa et al. 2017), in coincidence with the absence of fungicide cluster. The kernel bioassay, with findings similar to those observed in field conditions (Zhang et al. 2023), allowed us to observe variations in antifungal activities. The Bacillaceae family can control Fusarium head blight in wheat through both direct and indirect effects (Liu et al. 2023), but it is not possible to distinguish these mechanisms in these isolates due to the presence of pathways involved in both.

Greenhouse pot tests have been indicated as a key criterion for selecting plant growth-promoting bacteria (Akinrinlola et al. 2018). The exhaustive analysis carried out showed results consistent with the genomic, in vitro traits and documented capabilities of strains belonging to the *P. megaterium* and *B. velezensis* species (Chen et al. 2018; Ibarra-Villarreal et al. 2023; Liu et al. 2023). Both *B. velezensis* and three *P. megaterium* isolates produced significant changes in four or more different parameters related to growth promotion, with particularities that suggest in the mechanisms that employed. The strains *P. megaterium* ZAV-W64 and *B. velezensis* ZAV-W70 showed improvements in growth parameters generally associated with positive field performance, including increased root structure and aerial parameters. Particularly, root architecture, determined mainly by root length and the number of lateral roots, regulates plant adaptation to soil (Perotti et al. 2020). Additionally, an increase in root growth in pot and field assays has been reported in wheat treated with commercial strains of PGPR (Dal Cortivo et al. 2017). However, because discrepancies between pot assay results and field performance are common (Zeffa et al. 2020), field tests are essential for validating the efficacy of selected strains.

Numerous studies show that the Bacillaceae family can play important roles in disease control and yield increase in wheat, one of the most important cereal crops in the world, constantly challenged by biotic competitors and abiotic stresses (Liu et al. 2023). Even when commercial strains of Bacillaceae are applied as fertilizers and pesticides in wheat, these strains have mainly been isolated from soils and applied in environments differing from their native habitats, highlighting the necessity of generating novel isolates (Liu et al. 2023). It has been suggested that PGPR strains isolated from a region are better adapted to conditions prevalent in that region and, thus, would be more effective when applied to fields in the same region (Weller et al. 1985). The application of formulations based on the two best candidates improved yield, compared to the untreated control. ZAV-W70 produced increases in yield magnitude depending on whether the seeds were treated in combination with a fungicide in both assays, while ZAV-W64 only produced a significant increase in one of them. This increase could be associated with growth promotion capacity in both strains but also with antifungal capacity in ZAV-W70, as observed in the 2023 growing season. The discrepancies among seasons have been previously associated with environmental conditions, principally rainfall and temperatures during the crop cycle (Roy et al. 2020). Because grain yield is a complex trait resulting from multiplicative interactions among its components (Philipp et al. 2018), some approximations were made to explain the components modified by the isolates’ presence that led to the yield increase. Although increases in yield are sometimes associated with a loss of grain quality (Abeshu and Kasahun 2024), no such diminution was observed in this study. Moreover, seeds treated with ZAV-W64 in combination with fungicide exhibited improved bread-making quality, which is of great importance given the relevance of wheat in the human diet.

Finally, the results confirm the association between the described genetic and phenotypic traits observed, aligning with their higher abundance of beneficial traits related to plant growth promotion, in vitro assays, biocontrol, and plant growth promotion under controlled conditions. To the best of our knowledge, no studies have been carried out on bread wheat that involved a complete and precise genomic analysis and classification*,* in vitro and seedling analysis, and subsequent correlation with field assays and grain quality, spanning nearly all steps proposed from microorganism isolation to commercial production. These strains, with complementary genomic and functional traits, hold promise for developing sustainable wheat production technologies. Previous successes with microbial consortia in wheat production (Khan et al. 2022) support the exploration of combining ZAV-W70 and ZAV-W64 to further enhance agricultural sustainability without agrochemicals.

Supplementary information.

## Supplementary Information

Below is the link to the electronic supplementary material.

ESM 1 (PPTX 3.31 MB)

ESM 2 (PPTX 94.8 KB)

ESM 3 (PPTX 352 KB)

ESM 4 (PPTX 2.12 MB)

ESM 5 (XLSX 25.1 KB)

ESM 6 (XLSX 15.0 KB)

ESM 7 (DOCX 43.0 KB)

ESM 8 (XLSX 10.0 KB)

ESM 9 (DOCX 22.7 KB)

## Data Availability

The genome sequences generated during this study are available in the NCBI BioProject database under accession number PRJNA1234655. Additional data supporting the findings of this study are included in the manuscript and its supplementary information files. Strains ZAV-W70 and ZAV-W64 are deposited in the public culture collection of Acuario del Río Paraná (Universidad Nacional de Rosario, Santa Fe, Argentina) under the codes LBA104 and LBA111, respectively. They are available under Material Transfer Agreement (MTA) for non-commercial research.
