# Supplementary material for: Genomically-selected antifungal Bacillaceae strains improve wheat yield and baking quality
Source: Appl Microbiol Biotechnol. 2025 Jul 10;109:164. doi: 10.1007/s00253-025-13544-9 (PMC12241182; doi:10.1007/s00253-025-13544-9)
Supplement: Supplementary file 2 [file 253_2025_13544_MOESM9_ESM.docx]

**Supplementary Table S5A**: Yield Components and Growth Parameters of Wheat in the 2022 Growing Season.

| **Treatments** | | **Shoot dry weight (g)** | **P1000 grains (g)** | **Number of spikes / m^2^** | **Number of spikes / plants** | **Number of grains** |
| --- | --- | --- | --- | --- | --- | --- |
| Water | Water | 278.± 25 ab | 34.53 ± 0.12 a | 353 ±18 a | 1.49 ± 0.06 a | 3222 ± 486 ab |
|  | ZAV-W64 | 271 ± 33 ab | 34.51 ± 0.52 a | 463 ± 12 c | 2.02 ± 0.10 b | 3163 ± 560 ab |
|  | ZAV-W70 | 254 ± 12 a | 34.06 ± 0.73 a | 403 ± 31 b | 1.68 ± 0.17 a | 2927 ± 301 a |
| Fungicide | Water | 280 ± 26 ab | 36.58 ± 1.20 c | 421 ± 3 bc | 1.62 ± 0.10 a | 2958 ± 401 a |
|  | ZAV-W64 | 286 ± 11 ab | 35.81 ± 0.78 abc | 465 ± 8 c | 1.68 ± 0.08 a | 2802 ± 675 a |
|  | ZAV-W70 | 360 ± 21 c | 35.61 ± 0.36 ab | 421 ± 9 bc | 1.75 ± 0.07 ab | 3046 ± 80 b |

**Supplementary Table S5B**: Yield Components and Growth Parameters of Wheat in the 2023 Growing Season.

| **Treatments** | | **Moisture (%)** | **Plant height (cm)** | **Number of spikes / m^2^** | **P1000 grains (g)** |
| --- | --- | --- | --- | --- | --- |
| Water | Water | 12.15 ± 0.06 a | 52.75 ± 0.85 a | 256 ± 4 a | 39.58 ± 0.49 ab |
|  | ZAV-W64 | 12.08 ± 0.13 a | 53.25 ± 0.63 a | 257 ± 4 a | 39.23 ± 0.83 ab |
|  | ZAV-W70 | 11.90 ± 0.22 a | 56.50 ± 0.29 b | 291 ± 3 c | 37.65 ± 0.20 a |
| Fungicide | Water | 11.80 ± 0.20 a | 52.75 ± 0.25 a | 274 ± 2 b | 40.64 ± 0.16 b |
|  | ZAV-W64 | 12.05 ± 0.10 a | 52.75 ± 1.11 a | 260 ± 4 a | 38.22 ± 0.78 a |
|  | ZAV-W70 | 12.18 ± 0.17 a | 55.75 ± 0.75 b | 274 ± 4 b | 40.71 ± 1.30 b |

**Supplementary Table S5C**: Seed Quality Parameters and Bread-Making Properties of Wheat.

| **Treatments** | | **Ash (%)** | **Moisture (%)** | **Test Weight (Kg/hl)** | **Protein (%)** |
| --- | --- | --- | --- | --- | --- |
| Water | Water | 1.83 ± 0.01 c | 13.57 ± 0.08 a | 72.43 ± 0.63 ab | 10.63 ± 0.42 a |
|  | ZAV-W64 | 1.76 ± 0.01 a | 13.45 ± 0.08 a | 72.80 ± 1.00 ab | 10.30 ± 0.11 a |
|  | ZAV-W70 | 1.79 ± 0.02 abc | 13.44 ± 0.08 a | 72.80 ± 0.79 ab | 10.57 ± 0.20 a |
| Fungicide | Water | 1.79 ± 0.02 abc | 13.57 ± 0.05 a | 74.40 ± 0.58 b | 10.49 ± 0.15 a |
|  | ZAV-W64 | 1.81 ± 0.02 bc | 13.55 ± 0.05 a | 72.03 ± 0.52 a | 11.43 ± 0.11 b |
|  | ZAV-W70 | 1.78 ± 0.02 ab | 13.46 ± 0.11 a | 71.75 ± 0.49 a | 10.58 ± 0.27 a |
