# Supplementary material for: Genomically-selected antifungal Bacillaceae strains improve wheat yield and baking quality
Source: Appl Microbiol Biotechnol. 2025 Jul 10;109(1):164. doi: 10.1007/s00253-025-13544-9 (PMC12241182; doi:10.1007/s00253-025-13544-9)
Supplement: Supplementary file 1 — (PPTX 3.31 MB) [file 253_2025_13544_MOESM1_ESM.pptx]

## Slide 1
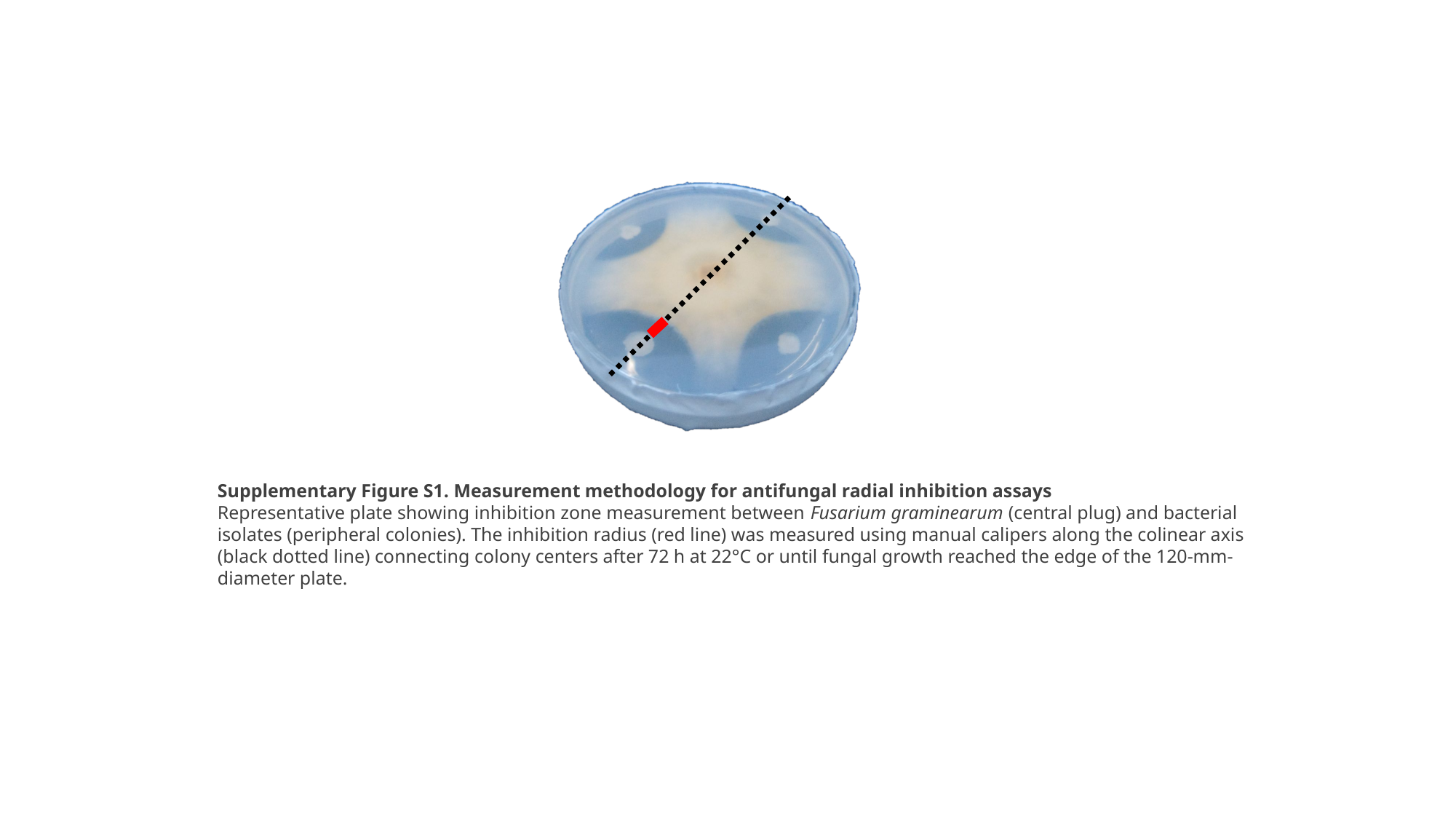

Supplementary Figure S1. Measurement methodology for antifungal radial inhibition assays
Representative plate showing inhibition zone measurement between Fusarium graminearum (central plug) and bacterial isolates (peripheral colonies). The inhibition radius (red line) was measured using manual calipers along the colinear axis (black dotted line) connecting colony centers after 72 h at 22°C or until fungal growth reached the edge of the 120-mm-diameter plate.
