# Supplementary material for: Genomically-selected antifungal Bacillaceae strains improve wheat yield and baking quality
Source: Appl Microbiol Biotechnol. 2025 Jul 10;109(1):164. doi: 10.1007/s00253-025-13544-9 (PMC12241182; doi:10.1007/s00253-025-13544-9)
Supplement: Supplementary file 2 — (PPTX 94.8 KB) [file 253_2025_13544_MOESM2_ESM.pptx]

## Slide 1
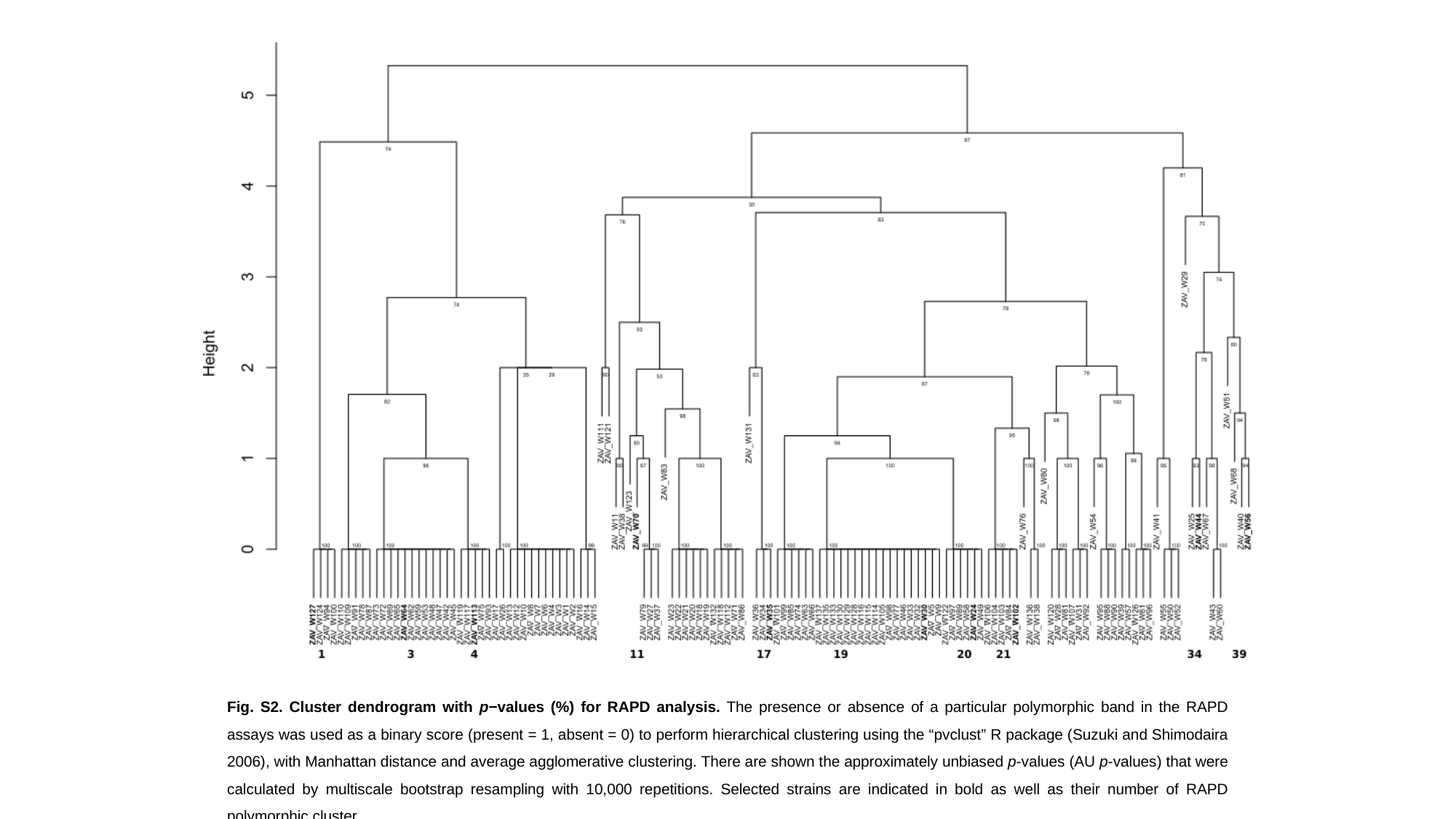

Fig. S2. Cluster dendrogram with p−values (%) for RAPD analysis. The presence or absence of a particular polymorphic band in the RAPD assays was used as a binary score (present = 1, absent = 0) to perform hierarchical clustering using the “pvclust” R package (Suzuki and Shimodaira 2006), with Manhattan distance and average agglomerative clustering. There are shown the approximately unbiased p-values (AU p-values) that were calculated by multiscale bootstrap resampling with 10,000 repetitions. Selected strains are indicated in bold as well as their number of RAPD polymorphic cluster.
