# Supplementary material for: Genomically-selected antifungal Bacillaceae strains improve wheat yield and baking quality
Source: Appl Microbiol Biotechnol. 2025 Jul 10;109(1):164. doi: 10.1007/s00253-025-13544-9 (PMC12241182; doi:10.1007/s00253-025-13544-9)
Supplement: Supplementary file 3 — (PPTX 352 KB) [file 253_2025_13544_MOESM3_ESM.pptx]

## Slide 1
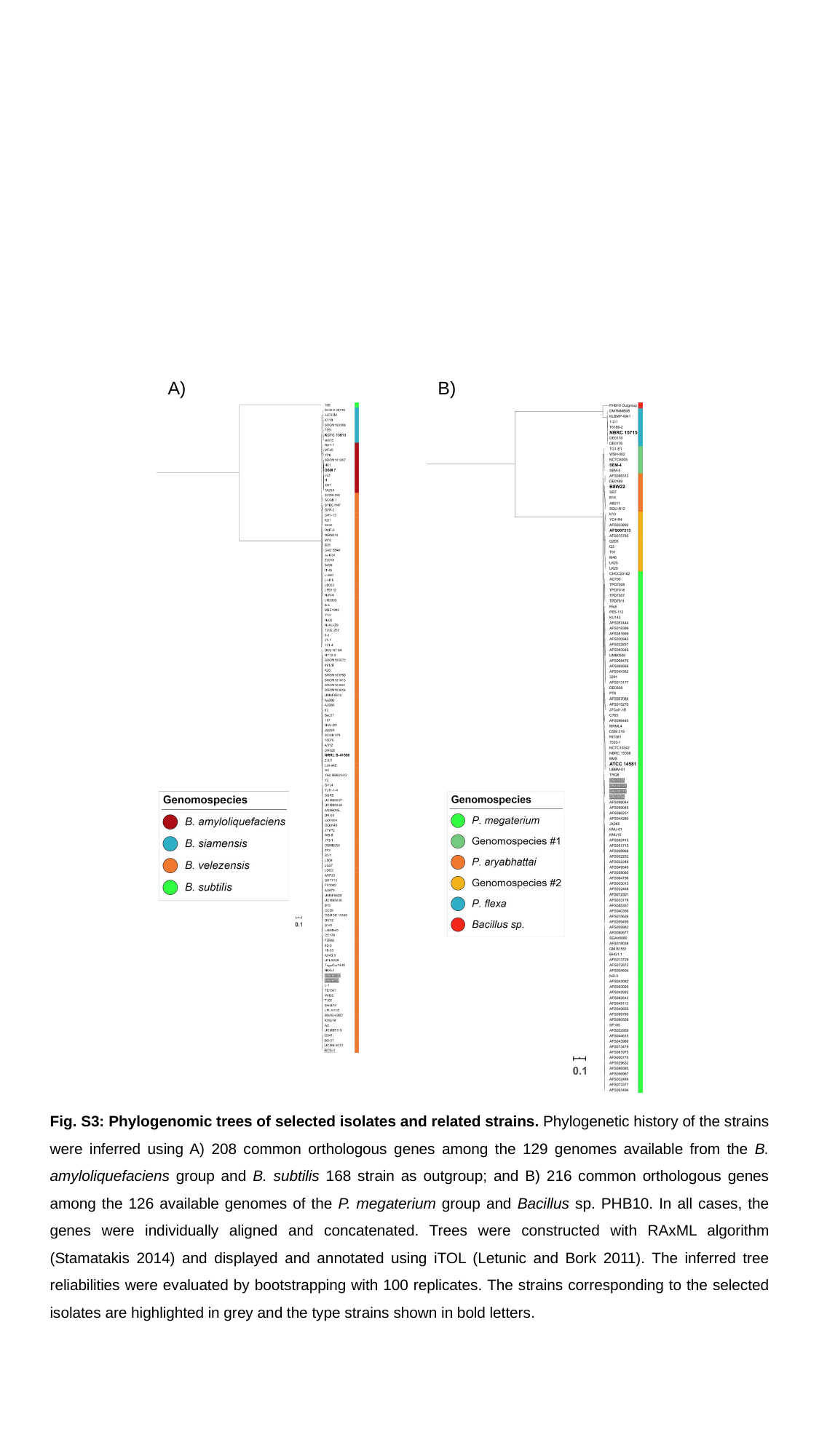

A)
B)
Fig. S3: Phylogenomic trees of selected isolates and related strains. Phylogenetic history of the strains were inferred using A) 208 common orthologous genes among the 129 genomes available from the B. amyloliquefaciens group and B. subtilis 168 strain as outgroup; and B) 216 common orthologous genes among the 126 available genomes of the P. megaterium group and Bacillus sp. PHB10. In all cases, the genes were individually aligned and concatenated. Trees were constructed with RAxML algorithm (Stamatakis 2014) and displayed and annotated using iTOL (Letunic and Bork 2011). The inferred tree reliabilities were evaluated by bootstrapping with 100 replicates. The strains corresponding to the selected isolates are highlighted in grey and the type strains shown in bold letters.
