# Supplementary material for: Genomically-selected antifungal Bacillaceae strains improve wheat yield and baking quality
Source: Appl Microbiol Biotechnol. 2025 Jul 10;109(1):164. doi: 10.1007/s00253-025-13544-9 (PMC12241182; doi:10.1007/s00253-025-13544-9)
Supplement: Supplementary file 4 — (PPTX 2.12 MB) [file 253_2025_13544_MOESM4_ESM.pptx]

## Slide 1
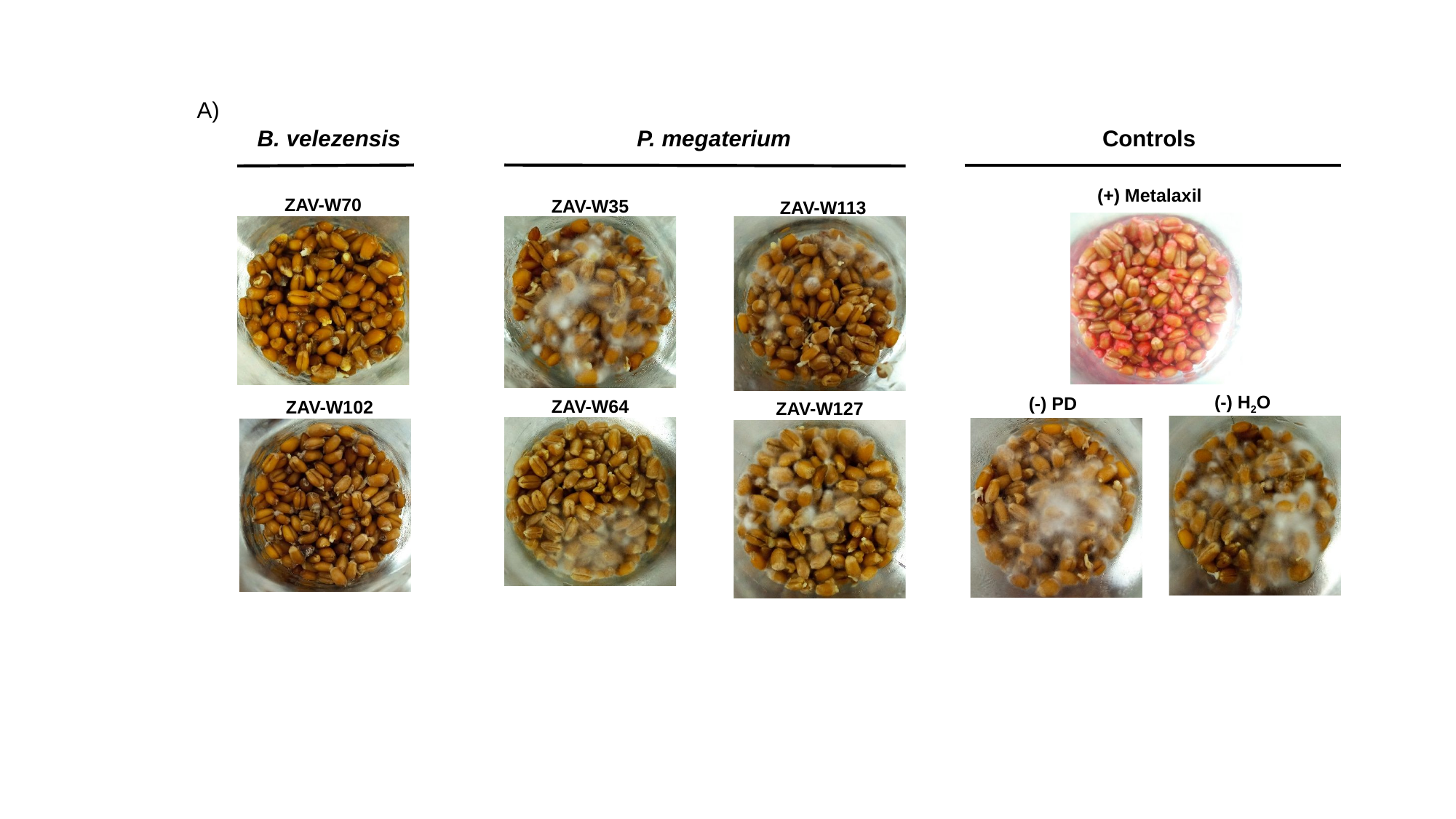

A)
B. velezensis
P. megaterium
Controls
(+) Metalaxil
ZAV-W70
ZAV-W35
ZAV-W113
(-) H2O
(-) PD
ZAV-W64
ZAV-W102
ZAV-W127

## Slide 2
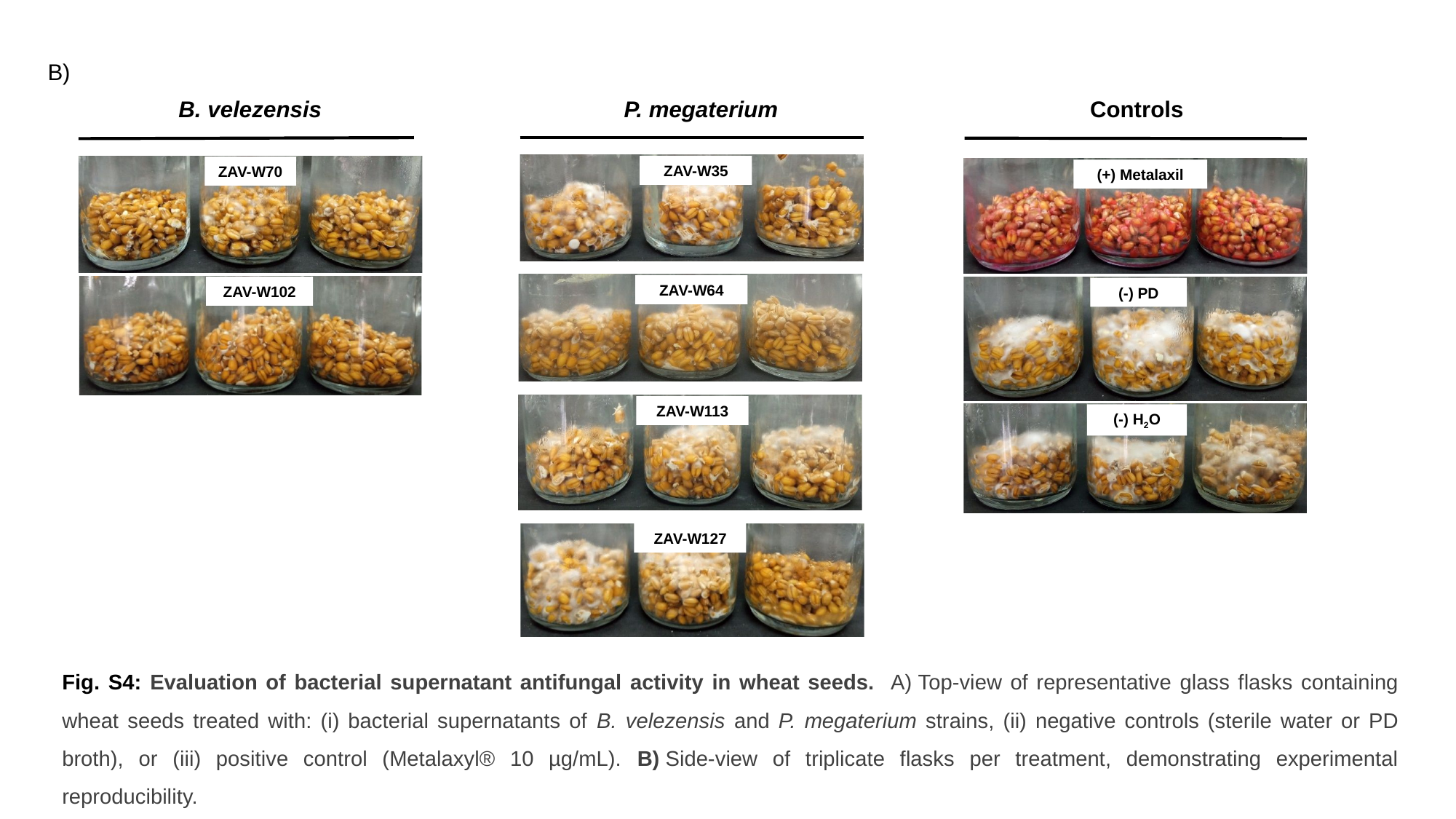

B)
B. velezensis
P. megaterium
Controls
ZAV-W35
ZAV-W70
ZAV-W102
(+) Metalaxil
(-) PD
(-) H2O
ZAV-W64
ZAV-W113
ZAV-W127
Fig. S4: Evaluation of bacterial supernatant antifungal activity in wheat seeds. A) Top-view of representative glass flasks containing wheat seeds treated with: (i) bacterial supernatants of B. velezensis and P. megaterium strains, (ii) negative controls (sterile water or PD broth), or (iii) positive control (Metalaxyl® 10 µg/mL). B) Side-view of triplicate flasks per treatment, demonstrating experimental reproducibility.
