# Supplementary material for: Genomically-selected antifungal Bacillaceae strains improve wheat yield and baking quality
Source: Appl Microbiol Biotechnol. 2025 Jul 10;109(1):164. doi: 10.1007/s00253-025-13544-9 (PMC12241182; doi:10.1007/s00253-025-13544-9)
Supplement: Supplementary file 7 — (DOCX 43.0 KB) [file 253_2025_13544_MOESM7_ESM.docx]

| **Table S3. Biosynthetic gene clusters and associated pathways in Bacillaceae strains** | | |
| --- | --- | --- |
| **Pathway** | **Genes** | **Reference** |
| associated with antifungal activities | | |
| Iturin A | *ituABCD* | (Dunlap et al., 2019) |
| Fengycin | *fenABCDE* | (Chen et al., 2008; Chen, Koumoutsi, et al., 2009a; Koumoutsi et al., 2004) |
| associated with antibacterial activities | | |
| Bacillaene | *baeBCDEGHIJLMNR* | (Chen, Koumoutsi, et al., 2009a) |
| Difficidin | *dfnABCDEFGHIJKLMXY* | (Chen, Scholz, et al., 2009; Wu et al., 2015) |
| Macrolactin | *mlnABCDEFGHI* | (Schneider et al., 2007) |
| Surfactin | *srfAA, srfAB, srfAC, srfAD* | (Chowdhury et al., 2015; Koumoutsi et al., 2004; Rahman & Randhawa, 2015) |
| Bacillysin | *bacABCDE* | (Chen, Scholz, et al., 2009; Wu et al., 2014, 2015) |
| Amylocyclicin | *acnBACDEF* | (Scholz et al., 2014) |
| Plantazolicin | *pnzFKGHAJCDBEL* | (Liu et al., 2013; Scholz et al., 2011) |
| associated with plant growth promotion | | |
| IAA | *ipdC, trpABDE, ysnE, dhaS, yhcX* | (Chen et al., 2007; Idris et al., 2007) |
| Acetoin | *alsDSR* | (Kierul et al., 2015; Ryu et al., 2003) |
| H_2_S | *cysNDIJCH* | (Mansilla & de Mendoza, 1997; van der Ploeg et al., 2001) |
| GABA | *speAB, puuABCD* | (Kim et al., 2023) |
| PAA | *ipdC* | (Idris et al., 2007) |
| Bacillibactin | *dhbABCDEF* | (Chen, Koumoutsi, et al., 2009a) |
| Lactonase | *aiiA* | (Yin et al., 2010) |
| Petrobactin | *asbABCDEF* | (Chen et al., 2007; Hagan et al., 2016) |
| Polyamine | *speABDE* | (Sekowska et al., 1998, 2000) |
| exoenzyme production | | |
| Amylase | *AmyE* | (Chen, Koumoutsi, et al., 2009a) |
| Cellulase | *bglACS* | (Zhang et al., 2015) |
| Xylanase | *XynAD* | (Chen, Koumoutsi, et al., 2009a) |
| Chitinase | *chi or chi113 or cbp50* | (Chen, Koumoutsi, et al., 2009a) |
| associated with other phenotypes | | |
| Biofilm | *tapA, pgcA, gtaB, epsG, epsE, sipW* | (Vlamakis et al., 2013) |

**Bibliography**

Chen, X. H., Koumoutsi, A., Scholz, R., & Borriss, R. (2008). More than anticipated—Production of antibiotics and other secondary metabolites by *Bacillus amyloliquefaciens* FZB42. *Journal of Molecular Microbiology and Biotechnology*, *16*(1-2), 14-24. https://doi.org/10.1159/000142891

Chen, X. H., Koumoutsi, A., Scholz, R., Eisenreich, A., Schneider, K., Heinemeyer, I., Morgenstern, B., Voss, B., Hess, W. R., Reva, O., Junge, H., Voigt, B., Jungblut, P. R., Vater, J., Süssmuth, R., Liesegang, H., Strittmatter, A., Gottschalk, G., & Borriss, R. (2007). Comparative analysis of the complete genome sequence of the plant growth–promoting bacterium *Bacillus* *amyloliquefaciens* FZB42. *Nature Biotechnology*, *25*(9), 1007-1014. https://doi.org/10.1038/nbt1325

Chen, X. H., Koumoutsi, A., Scholz, R., Schneider, K., Vater, J., Süssmuth, R., Piel, J., & Borriss, R. (2009). Genome analysis of *Bacillus amyloliquefaciens* FZB42 reveals its potential for biocontrol of plant pathogens. *Journal of Biotechnology*, *140*(1-2), 27-37. https://doi.org/10.1016/j.jbiotec.2008.10.011

Chen, X. H., Scholz, R., Borriss, M., Junge, H., Mögel, G., Kunz, S., & Borriss, R. (2009). Difficidin and bacilysin produced by plant-associated *Bacillus amyloliquefaciens* are efficient in controlling fire blight disease. *Journal of Biotechnology*, *140*(1-2), 38-44. https://doi.org/10.1016/j.jbiotec.2008.10.015

Chowdhury, S. P., Hartmann, A., Gao, X., & Borriss, R. (2015). Biocontrol mechanism by root-associated *Bacillus amyloliquefaciens* FZB42 – a review. *Frontiers in Microbiology*, *6*. https://doi.org/10.3389/fmicb.2015.00780

Dunlap, C. A., Bowman, M. J., & Rooney, A. P. (2019). Iturinic lipopeptide diversity in the *Bacillus subtilis* species group – important antifungals for plant disease biocontrol applications. *Frontiers in Microbiology*, *10*(AUG), 1-12. https://doi.org/10.3389/fmicb.2019.01794

Hagan, A. K., Carlson, P. E., & Hanna, P. C. (2016). Flying under the radar: The non‐canonical biochemistry and molecular biology of petrobactin from *Bacillus anthracis*. *Molecular Microbiology*, *102*(2), 196-206. https://doi.org/10.1111/mmi.13465

Idris, E. E., Iglesias, D. J., Talon, M., & Borriss, R. (2007). Tryptophan-Dependent Production of Indole-3-Acetic Acid (IAA) Affects Level of Plant Growth Promotion by *Bacillus amyloliquefaciens* FZB42. *Molecular Plant-Microbe Interactions®*, *20*(6), 619-626. https://doi.org/10.1094/MPMI-20-6-0619

Kierul, K., Voigt, B., Albrecht, D., Chen, X.-H., Carvalhais, L. C., & Borriss, R. (2015). Influence of root exudates on the extracellular proteome of the plant growth-promoting bacterium *Bacillus amyloliquefaciens* FZB42. *Microbiology*, *161*(1), 131-147. https://doi.org/10.1099/mic.0.083576-0

Kim, T., Heo, S., Na, H.-E., Lee, G., Lee, J.-H., Kim, J.-Y., & Jeong, D.-W. (2023). Increased Production of γ-Aminobutyric Acid from Brewer’s Spent Grain Through *Bacillus* Fermentation. *Journal of Microbiology and Biotechnology*, *33*(4), 527-532. https://doi.org/10.4014/jmb.2210.10051

Koumoutsi, A., Chen, X.-H., Henne, A., Liesegang, H., Hitzeroth, G., Franke, P., Vater, J., & Borriss, R. (2004). Structural and functional characterization of gene clusters directing nonribosomal synthesis of bioactive cyclic lipopeptides in *Bacillus amyloliquefaciens* strain FZB42. *Journal of Bacteriology*, *186*(4), 1084-1096. https://doi.org/10.1128/JB.186.4.1084-1096.2004

Liu, Z., Budiharjo, A., Wang, P., Shi, H., Fang, J., Borriss, R., Zhang, K., & Huang, X. (2013). The highly modified microcin peptide plantazolicin is associated with nematicidal activity of *Bacillus amyloliquefaciens* FZB42. *Applied Microbiology and Biotechnology*, *97*(23), 10081-10090. https://doi.org/10.1007/s00253-013-5247-5

Mansilla, M. C., & de Mendoza, D. (1997). L-cysteine biosynthesis in *Bacillus subtilis*: Identification, sequencing, and functional characterization of the gene coding for phosphoadenylylsulfate sulfotransferase. *Journal of Bacteriology*, *179*(3), 976-981. https://doi.org/10.1128/jb.179.3.976-981.1997

Rahman, P. K. S. M., & Randhawa, K. K. S. (2015). Editorial: Microbiotechnology Based Surfactants and Their Applications. *Frontiers in Microbiology*, *6*. https://doi.org/10.3389/fmicb.2015.01344

Ryu, C.-M., Farag, M. A., Hu, C.-H., Reddy, M. S., Wei, H.-X., Paré, P. W., & Kloepper, J. W. (2003). Bacterial volatiles promote growth in *Arabidopsis*. *Proceedings of the National Academy of Sciences*, *100*(8), 4927-4932. https://doi.org/10.1073/pnas.0730845100

Schneider, K., Chen, X.-H., Vater, J., Franke, P., Nicholson, G., Borriss, R., & Süssmuth, R. D. (2007). Macrolactin is the polyketide biosynthesis product of the *pks2* cluster of *Bacillus amyloliquefaciens* FZB42. *Journal of Natural Products*, *70*(9), 1417-1423. https://doi.org/10.1021/np070070k

Scholz, R., Molohon, K. J., Nachtigall, J., Vater, J., Markley, A. L., Süssmuth, R. D., Mitchell, D. A., & Borriss, R. (2011). Plantazolicin, a Novel Microcin B17/Streptolysin S-Like Natural Product from *Bacillus amyloliquefaciens* FZB42. *Journal of Bacteriology*, *193*(1), 215-224. https://doi.org/10.1128/JB.00784-10

Scholz, R., Vater, J., Budiharjo, A., Wang, Z., He, Y., Dietel, K., Schwecke, T., Herfort, S., Lasch, P., & Borriss, R. (2014). Amylocyclicin, a Novel Circular Bacteriocin Produced by *Bacillus amyloliquefaciens* FZB42. *Journal of Bacteriology*, *196*(10), 1842-1852. https://doi.org/10.1128/JB.01474-14

Sekowska, A., Bertin, P., & Danchin, A. (1998). Characterization of polyamine synthesis pathway in *Bacillus subtilis* 168. *Molecular Microbiology*, *29*(3), 851-858. https://doi.org/10.1046/j.1365-2958.1998.00979.x

Sekowska, A., Coppée, J., Le Caer, J., Martin‐Verstraete, I., & Danchin, A. (2000). S‐adenosylmethionine decarboxylase of *Bacillus subtilis* is closely related to archaebacterial counterparts. *Molecular Microbiology*, *36*(5), 1135-1147. https://doi.org/10.1046/j.1365-2958.2000.01930.x

van der Ploeg, J. R., Barone, M., & Leisinger, T. (2001). Functional analysis of the *Bacillus subtilis cysK* and *cysJI* genes. *FEMS Microbiology Letters*, *201*(1), 29-35. https://doi.org/10.1111/j.1574-6968.2001.tb10728.x

Vlamakis, H., Chai, Y., Beauregard, P., Losick, R., & Kolter, R. (2013). Sticking together: Building a biofilm the *Bacillus subtilis* way. *Nature Reviews Microbiology*, *11*(3), 157-168. https://doi.org/10.1038/nrmicro2960

Wu, L., Wu, H., Chen, L., Xie, S., Zang, H., Borriss, R., & Gao, X. (2014). Bacilysin from *Bacillus amyloliquefaciens* FZB42 Has Specific Bactericidal Activity against Harmful Algal Bloom Species. *Applied and Environmental Microbiology*, *80*(24), 7512-7520. https://doi.org/10.1128/AEM.02605-14

Wu, L., Wu, H., Chen, L., Yu, X., Borriss, R., & Gao, X. (2015). Difficidin and bacilysin from *Bacillus amyloliquefaciens* FZB42 have antibacterial activity against *Xanthomonas oryzae* rice pathogens. *Scientific Reports*, *5*(1), 12975. https://doi.org/10.1038/srep12975

Yin, X.-T., Xu, L., Fan, S.-S., Xu, L.-N., Li, D.-C., & Liu, Z.-Y. (2010). Isolation and characterization of an AHL lactonase gene from *Bacillus amyloliquefaciens*. *World Journal of Microbiology and Biotechnology*, *26*(8), 1361-1367. https://doi.org/10.1007/s11274-010-0308-8

Zhang, N., Yang, D., Wang, D., Miao, Y., Shao, J., Zhou, X., Xu, Z., Li, Q., Feng, H., Li, S., Shen, Q., & Zhang, R. (2015). Whole transcriptomic analysis of the plant-beneficial rhizobacterium *Bacillus amyloliquefaciens* SQR9 during enhanced biofilm formation regulated by maize root exudates. *BMC Genomics*, *16*(1), 685. https://doi.org/10.1186/s12864-015-1825-5
